# Organic Solvent-Free Olefins and Alcohols (ep)oxidation Using Recoverable Catalysts Based on [PM_12_O_40_]^3−^ (M = Mo or W) Ionically Grafted on Amino Functionalized Silica Nanobeads

**DOI:** 10.3390/ma12203278

**Published:** 2019-10-09

**Authors:** Yun Wang, Florence Gayet, Pascal Guillo, Dominique Agustin

**Affiliations:** 1 CNRS, LCC (Laboratoire de Chimie de Coordination), Université de Toulouse, UPS, INPT, 205, route de Narbonne, F-31077 Toulouse, France; thomaswang1990@hotmail.com (Y.W.); florence.gayet@ensiacet.fr (F.G.); pascal.guillo@iut-tlse3.fr (P.G.); 2 Université de Toulouse, Institut Universitaire de Technologie Paul Sabatier-Département de Chimie, Av. Georges Pompidou, BP 20258, F-81104 Castres, CEDEX, France; 3 INPT, ENSIACET 4, allée Emile Monso-CS 44362, F-31030 Toulouse, CEDEX 4, France

**Keywords:** catalysis, (ep)oxidation, silica beads, nanoparticles, functionalization, polyoxometalates, ionic grafting, organic solvent-free process

## Abstract

Catalyzed organic solvent-free (ep)oxidation were achieved using H_3_PM_12_O_40_ (M = Mo or W) complexes ionically grafted on APTES-functionalized nano-silica beads obtained from straightforward method (APTES = aminopropyltriethoxysilane). Those catalysts have been extensively analyzed through morphological studies (Dynamic Light Scattering (DLS), TEM) and several spectroscopic qualitative (IR, multinuclear solid-state NMR) and quantitative (^1^H and ^31^P solution NMR) methods. Interesting catalytic results were obtained for the epoxidation of cyclooctene, cyclohexene, limonene and oxidation of cyclohexanol with a lower [POM]/olefin ratio. The catalysts were found to be recyclable and reused during three runs with similar catalytic performances.

## 1. Introduction

Oxidation of olefins and alcohols are important reactions in organic chemistry, with fundamental and applicative interest of those chemical transformations [1,2,3,4,5,6,7,8,9,10]. Indeed, epoxides can be obtained using very efficient organic oxidants (*m*-CPBA [11], NaClO [12] and RCO_3_H [13,14]) but with procedures needing long workup, detrimental for ecological and economical purposes. Those issues can be diminished using metal-based catalysts, but some drawbacks of those processes come from the use of (toxic) metals and/or the use of (toxic) organic solvent(s). As examples for epoxidation, several Mo complexes used in industrial plants need the use of chlorinated solvents [15] such as 1,2-dichloroethane (DCE), a highly toxic solvent that has to be avoided [16]. An elegant way to circumvent those issues, in straight line with the principles of green chemistry [17,18,19], is to diminish/suppress the use of organic solvent within the oxidation process. We demonstrated it in several organic solvent-free processes we published with active (pre)catalysts (Mo, V or W based, complexes with tridentate ligands and/or polyoxometalates (POMs)), giving a first step towards a cleaner process [20,21,22,23,24,25,26,27,28,29,30,31]. In term of an efficient greener process, the best oxidant found with our protocols was TBHP in aqueous solution for the epoxidation since the “waste”, tBuOH is potentially recycled [32]. In terms of atom economy, the oxidation reactions could be even more improved using for example H_2_O_2_ or O_2_ as oxidant [33]. Although advantageous, some of those cited processes could not allow an easy separation of the catalysts from the media. One strategy we employed to recover the POM catalyst was to graft ionically the catalyst on Merrifield resin [34]. Merrifield resin as covalent support has been used by other research groups especially with vanadium [35]. Other supports were also explored [36]. Mesoporous silica as a support was tried by several research groups in order to retain the POMs within the pores [37,38,39,40,41]. Although the compounds were active (under organic solvent conditions mainly), the disadvantage of mesoporous supports is the potential incomplete accessibility of the POMs present in the material for a catalytic process and thus a loss of efficiency. For this, it was interesting to take advantage to the known sol–gel chemistry and Stöber process, and the possibility of functionalization of silica—using trialkoxysilane precursors—to obtain pending ammonium functions only on the surface of the silica beads [42,43,44]. This strategy was employed for different uses, in order to graft in a covalent way polydentate ligands and related complexes or POMs for catalyzed reactions [45,46], luminescence properties [47,48], analytical purposes [49], to trap heavy metals for depollution [50] concerns or organic molecules for controlled release [51], mainly on mesoporous compounds [52,53] but scarcely with non-porous silica beads [54,55,56,57]. In order to find an easy recoverable catalyst, those functionalized silica beads played the role of countercations of the anionic polyoxometalate. The influence of the metal (Mo vs. W) was studied on different substrates. In the case of the most active complex, the silica beads were recovered and reused three times.

## 2. Materials and Methods 

### 2.1. Materials 

All manipulations were carried out under air. Distilled water was used directly from a Milli-Q purification system (Millipore, Burlington, MA, USA). Acetonitrile, ethanol, methanol and diethyl ether (synthesis grade, Aldrich, St. Louis, MI, USA) were used as solvents and employed as received. Tetraethyl orthosilicate (TEOS, 98% Aldrich), ammonium hydroxide solution (25%, Aldrich), 3-aminopropyltriethoxysilane (APTES, 99%, Aldrich), *cis*-cyclooctene (CO, 95%, Alfa Aesar, Ward Hill, MA, USA), cyclooctene oxide (COE, 99%, Aldrich), cyclohexene (CH, 99%, Acros, Geel, Belgium), cyclohexene oxide (CHO, 98%, Aldrich), 2-cyclohexen-1-ol (CHol, 95%, TCI, Tokyo, Japan), 2-cyclohexen-1-one (CHone, 96%, TCI), *cis*-1,2-cyclohexanediol (CHD, 99%, Acros), limonene (Lim, 98%, Aldrich), limonene oxide (LO *cis*/*trans* mixture, 97%, Aldrich), (1S, 2S, 4R)-(+)-limonene-1,2-diol (*ax*-LD, 97%, Aldrich), L-carveol (C^ol^ *cis*/*trans* mixture, 95%, Aldrich), (R)-(-) Carvone (C^one^ 98%, Aldrich), cyclohexanol (CYol, 99%, Alfa Aesar, Karlsruhe, Germany), cyclohexanone (CYone, 99.8%, Acros), phosphotungstic acid hydrate (reagent grade, Aldrich), molybdatophosphoric acid hydrate (reagent grade, Merck, Darmstadt, Germany) and TBHP (70% in water, Aldrich) were used as received. The pure *cis*-LO, *trans*-LO and *eq*-LD were synthesized according to literature procedures [58,59,60]. 

### 2.2. Methods

Powder X-ray diffraction: The solids were analyzed by X-ray diffraction (XRD) with a Bruker (Karlsruhe, Germany) D2 X’Pert PRO diffractometer using Cu Kα radiation (40 kV and 40 mA).

Dynamic Light Scattering (DLS): In order to be able to obtain repetitive and correct data analysis, particle samples were prepared at 0.1 wt.% in water. Sonication of particles suspension was made before DLS analysis during 10 min at 350 W (FB705 Fisherbrand Ultrasonic Processor, Fisherbrand, Waltham, MA, US) and using an ice bath, facilitating the dispersion of silica particles. Hydrodynamic diameters of the particles in suspension were obtained with a ZetaSizer Nano-ZS (Malvern Instruments Ltd., Worcestershire, UK,) at 25 °C. This equipment uses a laser (He-Ne at λ = 633 nm, under voltage of 3 mV) and the detector was located at 173° to analyze the scattered intensity fluctuations.

TEM: Particles morphology was performed with a JEOL (Tokyo, Japan) JEM1400 transmission electron microscope equipped with 120 kV voltage acceleration and tungsten filament (Service Commun de Microscopie Electronique TEMSCAN, Centre de Microcaractérisation Raimond Castaing, Toulouse, France). A drop of sonicated particles solution (at 0.1 wt.% in water) was disposed on a formvar/carbon-coated copper grid (400 mesh) and dried in air for 48 h.

Infrared spectroscopy: Fourier transform infrared (FTIR) spectra were recorded by Spectrum two—PerkinElmer (Llantrisant, UK).

Solid state NMR: NMR experiments were recorded on Bruker Avance (Fällanden, Switzerland) 400 III HD spectrometers operating at magnetic fields of 9.4 T. Samples were packed into 4 mm zirconia rotors. The rotors were spun at 8 kHz at 293 K. ^1^H MAS was performed with DEPTH pulse sequence and a relaxation delay of 3 s. For ^29^Si MAS single pulse experiments, small flip angle of 30° were used with a recycle delays of 60 s. ^13^C CP and ^29^Si CP MAS spectra were recorded with a recycle delay of 2 s and contact times of 3 ms and 4 ms, respectively. Chemical shifts were referenced to TMS. All spectra were fitted using the DMfit software (version 20190125).

Solution NMR: ^1^H-NMR, ^13^C-NMR and ^31^P-NMR spectra were recorded on Bruker (Fällanden, Switzerland) NMR III HD 400 MHz spectrometers. 400 MHz for ^1^H-NMR, 101 MHz for ^13^C-NMR and 162 MHz for ^31^P-NMR.

Quantification of the number of functions per gram of grafted silica through ^1^H NMR in solution: 7 mg of SiO_2_@R (R=NH_2_, PM) were added in 4 mL of D_2_O/NaOH solution (pH ≈ 13) in an NMR tube. The mixture was heated until the powder completely dissolved. A known amount of benzoic acid (ca. 4 mg) was added as internal standard. Then the NMR proton data were collected immediately.

Quantification of the number of POMs per gram of grafted silica through ^31^P NMR in solution: 7 mg of SiO_2_@PM was added in 4 mL of D_2_O/NaOH solution (pH ≈ 13) in an NMR tube. The mixture was heated until the powder completely dissolved. The PM amount per gram of the SiO_2_@PM sample was calculated from calibration curves (r^2^ = 0.999) obtained with different concentrations on phosphates at the same pH. 

Elemental analysis: Elemental analyses (EA) were performed by the LCC microanalysis service on PerkinElmer 2400 série II (Llatrisant, UK) 

Centrifugation: The silica beads were collected by centrifugation on a Sigma 2-16P with 11192 rotor (Max. rpm 4500, Sigma, Osterode am Harz, Germany).

Gas chromatography: The catalytic reactions were followed by gas chromatography (GC) on an Agilent 7820A chromatograph equipped with an FID detector, a DB-WAX capillary column (30 m × 0.32 mm × 0.5 μm) and autosampler. Authentic samples of reactants (cyclooctene, cyclohexene, limonene and cyclohexanol) and some potential products (cyclooctene oxide, cyclohexene oxide, 2-cyclohexen-1-ol, *cis*-1.2-cyclohexanediol, 2-cyclohexen-1-ol, L-carveol, R-carvone, limonene oxide, limonene-diol and cyclohexanone) were used for calibration. The Lim conversion and the formation of LimOs and limDs were calculated from the calibration curves (r^2^ = 1) and an internal standard.

### 2.3. Synthesis of Nanoparticles.

Synthesis of Stöber SiO_2_ nanoparticles.

72 mL of H_2_O (4 mol) and 60 mL of ammonic solution (28% wt) were mixed in 630 mL (15.57 mol) of methanol at room temperature. 40 mL of tetraethyl orthosilicate (TEOS) (0.18 mol) were added into the solution. A suspension of a white solid appeared. The mixture was stirred at 50 °C for 6 h. Then the solid was washed by absolute ethanol five times and was collected by centrifugation. SiO_2_ nanoparticles were dried under vacuum at 120 °C overnight. A white powder was obtained.

SiO_2_: ^1^H NMR (400 MHz, D_2_O/NaOH-Benzoic acid) δ 7.65 (m, 2H, Ar–H), 7.29 (m, 3H, Ar–H) and 3.10 (s, 3H, CH_3_). ^29^Si CP MAS-NMR: –93.3 ppm (Q^2^), –101.9 ppm (Q^3^) and –111.8 ppm (Q^4^). EA. Found: C, 1.09%; H, 0.67%. IR (ATR, ν(cm^-1^)): 2930–3707 (OH), 1053 (Si–O–Si), 942 (Si–OH), 795 and 434 (Si–O–Si).

Synthesis of SiO_2_@NH_2_ particles.

3.5 g of SiO_2_ particles were mixed with 12.08 mL (51.6 mmol) of APTES in 87.5 mL (823.3 mmol) of toluene. The mixture was refluxed under stirring for 18h. The product was washed by toluene (5 mL × 40 mL) and collected by centrifugation. The collected powder was dried under vacuum at 120 °C overnight.

SiO_2_@NH_2_: ^1^H NMR for the quantification (400 MHz, D_2_O/NaOH-Benzoic acid) δ 7.6 (m, 2H, Ar–H), 7.25 (m, 3H, Ar–H), 3.33 (q, *J* = 7.1 Hz, 0.07H, CH_2_), 3.03 (s, 0.09H, CH_3_), 2.25 (t, *J* = 7.0 Hz 0.21H, CH_2_), 1.18 (m, 0.23H, CH_2_), 0.87 (t, *J* = 7.1 Hz 0.1H, CH_3_) and 0.08 (m, 0.23H, CH_2_). ^29^Si CP MAS-NMR: –62.1 ppm (T^2^), –67.7 ppm (T^3^), –92.8 ppm (Q^2^), –102.0 ppm (Q^3^) and –111.5 ppm (Q^4^). ^13^C CP MAS-NMR: 60.4 ppm (CH_2_O), 58.2 ppm (CH_2_O), 50.9 ppm (CH_2_N), 42.3 ppm (CH_2_N), 21.5 ppm (CH_2_), 16.5 ppm (CH_3_) and 9.6 ppm (CH_2_Si). EA. Found: C, 3.74%; H, 1.32% and N, 0.74%. IR (ATR, ν(cm^-1^)): 2969–3700 (OH and NH), 1485 (CH_2_ and NH_2_), 1049 (Si–O–Si), 939 (Si–OH), 785 and 429 (Si–O–Si). ρ(NH_2_) (mmol/g) = 0.52 (by ^1^H NMR) and 0.53 (by EA).

Syntheses of SiO_2_@PM objects.

500 mg of SiO_2_@NH_2_ and 0.32 g (0.1 mmol) of H_3_PW_12_O_40_–15H_2_O (0.23 g for H_3_PMo_12_O_40_–26H_2_O) were mixed in 10 mL of H_2_O at 60 °C and stirred for 24 h. The product was washed by H_2_O (3 mL × 40 mL), collected by centrifugation as a powder and dried under vacuum at 120 °C overnight.

SiO_2_@PW: ^1^H NMR for the quantification (400 MHz, D_2_O/NaOH–benzoic acid) δ 7.66 (m, 2H, Ar–H), 7.31 (m, 3H, Ar–H), 3.41 (q, J = 7.1 Hz, 0.11H, CH_2_), 3.10 (s, 0.09H, CH_3_), 2.33 (t, J = 7.0 Hz, 0.18H, CH_2_), 1.26 (m, 0.18H, CH_2_), 0.94 (t, J = 7.1 Hz, 0.16H, CH_3_) and 0.17 (m, 0.19H, CH_2_). ^29^Si CP MAS-NMR: –58.3 ppm (T^2^), –67.9 ppm (T^3^), –93.0 ppm (Q^2^), –102.1 ppm (Q^3^) and –111.7 ppm (Q^4^). ^13^C CP MAS-NMR: 59.9 ppm (CH_2_O), 58.2 ppm (CH_2_O), 50.8 ppm (CH_2_N), 42.8 ppm (CH_2_N), 20.6 ppm (CH_2_), 16.6 ppm (CH_3_) and 9.2 ppm (CH_2_Si). ^31^P CP MAS-NMR: –12.8 ppm. IR (ATR, ν(cm^-1^)): 2969–3700 (–OH and –NH), 1485 (CH_2_ and NH_2_), 1065 (P–O), 1067 (Si–O–Si), 981 (W–O), 873 (W–O-W), 785 and 429 (Si–O–Si). EA. Found: C, 2.99%; H, 0.89% and N, 0.43%. ρ(NH_2_) (mmol/g) = 0.33 (by ^1^H NMR) and 0.31 (by EA). ρ(PW_12_) = 0.15 (by EA) and 0.14 (by ^31^P NMR).

SiO_2_@PMo: ^1^H NMR for the quantification (400 MHz, D_2_O/NaOH-Benzoic acid) δ 7.65 (m, 2H, Ar–H), 7.30 (m, 3H, Ar–H), 3.41 (q, *J* = 7.1 Hz, 0.16H, CH_2_), 3.10 (s, 0.13H, CH_3_), 2.33 (t, *J* = 7.0 Hz, 0.25H, CH_2_), 1.26 (m, 0.28H, CH_2_), 0.94 (t, *J* = 7.1 Hz, 0.23H, CH_3_) and 0.17 (m, 0.27H, CH_2_). ^29^Si CP MAS-NMR: –58.6 ppm (T^2^), –68.2 ppm (T^3^), –93 ppm (Q^2^), –102.1 ppm (Q^3^) and –111.7 ppm (Q^4^). ^13^C CP MAS-NMR: 59.9 ppm (CH_2_O), 58.2 ppm (CH_2_O), 50.9 ppm (CH_2_N), 42.9 ppm (CH_2_N), 20.7 ppm (CH_2_), 16.6 ppm (CH_3_) and 8.8 ppm (CH_2_Si). ^31^P CP MAS-NMR: –1.5 ppm. IR (ATR, ν(cm^-1^)): 2969–3700 (–OH and –NH), 1485 (CH_2_ and NH_2_), 1065 (P–O), 1068 (Si–O–Si), 944 (Mo–O), 879 (Mo–O–Mo), 785 and 443 (Si–O–Si). EA. Found: C, 2.49%; H, 1.29% and N, 0.59%. ρ(NH_2_) (mmol/g) = 0.40 (by ^1^H NMR) and 0.41 (by EA). ρ(PMo_12_) = 0.12 (by EA) and 0.12 (by ^31^P NMR).

### 2.4. Catalytic Experiments

Epoxidation of cyclooctene.

1.09 g (9.89 mmol) of cyclooctene, a quantity of catalyst (24.6 mg (7.8 μmol) of H_3_PW_12_O_40_–15H_2_O or 13.2 mg (5.7 μmol) of H_3_PMo_12_O_40_–26H_2_O or 50 mg of SiO_2_@PM (7.7 μmol for PW_12_ 5.7 μmol for PMo_12_)) and 0.35 mL (2.83 mmol) of acetophenone (internal standard) were mixed in a 25 mL flask. 2.05 mL of TBHP (14.84 mmol; 70 wt.% in H_2_O) were added into the mixture when the temperature was stabilized at 80 °C. The reaction mixture was heated at 80 °C under stirring for 24 h. The reaction was followed by GC-FID.

Epoxidation of cyclohexene.

With free-POM: 5 g (60.9 mmol) of cyclohexene were mixed with 26.9 mg (8.5 μmol) H_3_PW_12_O_40_. 15H_2_O (or 15.4 mg (6.7 μmol) for H_3_PMo_12_O_40_.26H_2_O) and 0.7 mL (5.66 mmol) of acetophenone (internal standard). 12.5 mL of TBHP (91.3 mmol; 70 wt.% in H_2_O) were added into the mixture at 60 °C and the solution was left under stirring for 48 h. The reaction was followed by GC-FID.

With SiO_2_@POM: 6.3 g (77 mmol) of cyclohexene, 75 mg SiO_2_@PM (11.5 μmol for PW_12_, 8.14 μmol for PMo_12_) and 0.7 mL (5.66 mmol) of acetophenone (internal standard) were mixed in a 50 mL flask. 15.2 mL of TBHP (111.3 mmol; 70 wt.% in H_2_O) were added into the mixture at 60 °C and left under stirring for 48 h. The reaction was followed by GC-FID.

Epoxidation of limonene.

2 g (14.9 mmol) of limonene, 33.3 mg (10.4 μmol) of H_3_PW_12_O_40_.15H_2_O or 19.6 mg (8.6 μmol) of H_3_PMo_12_O_40_.26H_2_O) or 75 mg of SiO_2_@PM (11.5 μmol for PW_12_, 8.6 μmol for PMo_12_) and 0.7 mL (5.66 mmol) of acetophenone (internal standard) were mixed in a 50 mL flask. 3.05 mL (22.3 mmol) of TBHP (70 wt.% in H_2_O) were added into the mixture at 80 °C. Then the mixture was heated at 80 °C under stirring for 24 h. The reaction was followed by GC-FID.

Oxidation of cyclohexanol.

With free-POM: 2 g (20 mmol) of cyclohexanol, 49.7 mg (14 μmol) of H_3_PW_12_O_40_–15H_2_O (23.5 mg (11.6 μmol) for H_3_PMo_12_O_40_–26H_2_O) and 0.35 mL (2.83 mmol) of acetophenone (internal standard) were mixed in a 50 mL flask. Of TBHP 4.1 mL (29.9 mmol; 70 wt.% in H_2_O) were added into the mixture at 80 °C. Then the mixture was heated at 80 °C under stirring for 24 h. The reaction was followed by GC-FID.

With SiO_2_@PM: 1.46 g (14.6 mmol) of cyclohexanol were mixed with 75 mg of SiO_2_@POM (11.52 μmol of POM for SiO_2_@PW and 8.6 μmol of POM for SiO_2_@PMo_12_) and 0.35 mL (2.83 mmol) of acetophenone (internal standard). Then 3 mL (21.9 mmol) of TBHP (70 wt.% in H_2_O) were added into the mixture at 80 °C. Then the mixture was stirred at 80 °C for 48 h. The reaction was followed by GC-FID.

## 3. Results and Discussion

### 3.1. Synthesis of the Catalytic Objects.

**Scheme 1:**
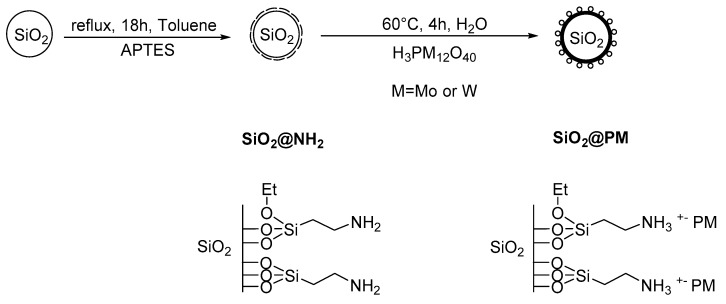
Schematic synthetic pathway of the different silica particles.

The synthesis of the catalytic objects was a three-step method (Scheme 1), starting from the synthesis of non-porous SiO_2_ beads according to a modified Stöber method using Si(OEt)_4_ (TEOS) and ammonia in MeOH [61]. The second step, i.e., the grafting of aminopropyltrietoxysilane (APTES) at the surface of SiO_2_, was performed under classical conditions in toluene [62], the grafted species with pending NH_2_ functions (named here SiO_2_@NH_2_) being isolated as white powder. The final step consisted of the ionic grafting of the POM catalysts on SiO_2_@NH_2_ simply by mixing in water SiO_2_@NH_2_ beads and the corresponding Keggin heteropolyacids H_3_PM_12_O_40_ (M=Mo or W) in water, in a POM/NH_2_ functions ratio of 1/3. The final solids SiO_2_@PMo and SiO_2_@PW were isolated as powders. In the case of molybdenum, the powder was slightly blue, indicating an interaction with ammonium [63]. The mixture with the tungsten gave a white powder.

Amorphous nature, as well as the sizes and morphologies of the isolated objects SiO_2_, SiO_2_@NH_2_, SiO_2_@PMo and SiO_2_@PW were analyzed before the catalytic experiments by PXRD, DLS and TEM. Accurate analysis of the functional content has been performed using IR and multinuclear solid-state NMR. Qualitative studies were performed through elemental analysis and ^1^H and ^31^P solution NMR.

### 3.2. Morphological Characterization of the Silica Beads

#### 3.2.1. Analysis by PXRD

The amorphous state of isolated SiO_2_@PM beads was characterized through powder X-ray diffraction (Figure S1) and did correspond to what was expected with 2θ = 23° [49,64,65]. At the difference with POMs grafted in a similar way in mesoporous SBA-15 [66], no diffraction peaks corresponding to the starting heteropolyacids (Figure S2) could be detected (in comparison with the PXRD spectra of the starting materials), certainly indicating that POMs were grafted but did not remain agglomerated in a crystalline way.

#### 3.2.2. Dynamic Light Scattering (DLS) Analysis.

**Figure 1:**
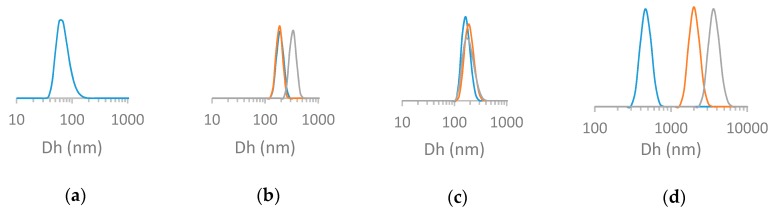
Size (hydrodynamic radius) distribution (in number) obtained by DLS for the SiO_2_ (**a**) SiO_2_@NH_2_ (**b**) SiO_2_@PW (**c**) and SiO_2_@PMo (**d**) beads. The time dependent size increase (case **b**–**d**) has been indicated by the different colors (blue-orange-grey).

The DLS measurements are usually performed to determine the hydrodynamic diameter of colloidal particles. As described previously with the silica particles obtained by the Stöber method [61], we considered the objects as spherical. This technique can give a perfect size (hydrodynamic diameter D_h_) of the particles when enough dispersed in the suspension and that no time depending aggregation phenomena do occur [67]. Measurements were performed with SiO_2_, SiO_2_@NH_2_, SiO_2_@PW and SiO_2_@PMo. The results have been graphically indicated in Figure 1.

The DLS measurements gave stable measurements within the time range for the SiO_2_ beads only (Figure 1a), the D_h_ found being around 70 nm in suspension in water. The SiO_2_@NH_2_ beads show different behavior (Figure 1b). Within the time, the size distribution was changing and aggregation seemed to occur, the diameter evolving from 190 nm at the first measurement (implicating some small association of the beads under the conditions of the measurements if we considered that the starting beads used for the grafting were the SiO_2_ presented previously) to even bigger aggregation with higher Rh values, i.e., 340 nm after a longer time. This might be due to the nature of the pending NH_2_ functions and the possibility of hydrogen bonds.

Addition of the POMs to the SiO_2_@NH_2_ beads did profound changes to the DLS measurements according to the nature of the POM. With SiO_2_@PW, the starting measured size was around 190 nm (as for the SiO_2_@NH_2_) and evolved to 220 nm (Figure 1c). In the case of the SiO_2_@PMo beads (Figure 1d), the starting value was huge from 450 nm to 3900 nm between the different time measurements. This was proof of a time-dependent rearrangement of the beads. DLS did not give information for the hydrodynamic radii of the single particles in this case but pointed out an aggregation phenomenon due to the nature of the surrounding of the silica particles, once the grafting was done. Interactions with the POMs (and protonation of the pending NH_2_) might change the pH value and favor the aggregation [68]. Such a phenomenon was observed with thiolated silica particles interacting with different concentrations of hydroxyethylcellulose [69].

#### 3.2.3. TEM Analysis

**Figure 2:**
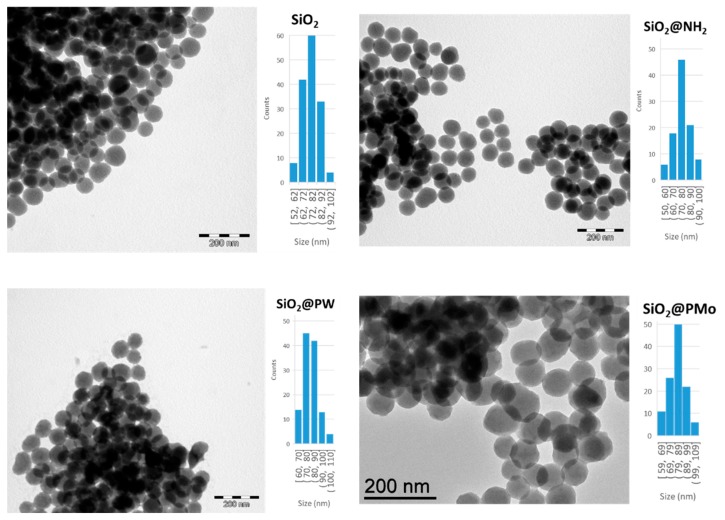
TEM images and diameters distribution of SiO_2_, SiO_2_@NH_2_, SiO_2_@PW and SiO_2_@PMo beads.

TEM measurements gave the proof of narrow dispersity of the silica beads (Figure 2). The beads had an average diameter of 75.9 nm for SiO_2_ and SiO_2_@NH_2_ and around 80.6 and 82.9 nm for the SiO_2_@PW and SiO_2_@PMo ones respectively, indicating that the structure of the SiO_2_ core was maintained during the three steps. The coverage of SiO_2_@NH_2_ with POMs could be proven in addition by a textural change of the surface of the beads.

**Figure 3:**
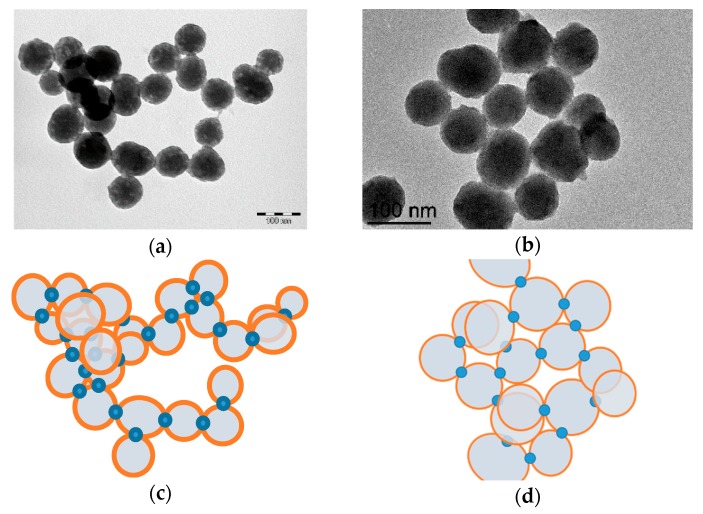
TEM images of SiO_2_@PW (**a**) and SiO_2_@PMo (**b**). Relative schematic potential interactions (**c**,**d**) are represented with blue circles.

Interesting observations could be done concerning the interactions between particles in the case of SiO_2_@PW and SiO_2_@PMo. The contrast at the contact area between particles in TEM pictures (Figure 3) seem to indicate contacts between the beads, stronger than with SiO_2_ or SiO_2_@NH_2_ with angles that seem to be not due to a simple packing. This feature could be similar to the one observed with Europium-containing POMs entrapped within SiO_2_ [70] on which fusing could be possible since silica covered the POMs but the situation described herein was reverse since POMs covered the surface of the silica beads. The first possible explanation could be that the surface of the beads was rearranged in the presence of acidic POM (and water media) twinning the silica nanoparticles. The surface of silica could be corroded and reacted again, fusing on contact points. This explanation could be valid since this phenomenon did need the presence of POMs and it was not observed in the case of SiO_2_@NH_2_. The fusing was proved by Greasley et al. using SiO_2_ particles and CaO [71] at temperatures higher than the ones used herein. Another explanation could be that POMs are “agglomerated” between SiO_2_ beads (but not in a crystalline arrangement since no peaks of the POMs were observed in the XRD spectra of SiO_2_@PM) and favor a simple ionic contact/fusing between the beads. This plausible explanation looks like silica beads functionalized with β-cyclodextrin and G1 adamantly PPI dendrimers (H-bond interactions) [72] or gold nanoparticles decorated with POMs (ionic interactions) [73]. Due to the nature of the silica part in SiO_2_@PM beads (positively charged in surface through ammonium functions), a complex association composed of POM/ammonium attractions and ammonium/ammonium repulsions might favor electrostatic interactions with specific angles corresponding to superficial contacts, the closest contact between three beads giving a triangular aspect. Thus, an explanation of the observed phenomenon in TEM seems to be situated between beads fusing and/or strong inter-bead electrostatic interactions. Both phenomena being possible in the aqueous media, it might be concluded that ionic rearrangements can occur when the species are mixed in water. Although TEM did give an image of a dried sample, the time dependent aggregation phenomena observed in solution through DLS seemed to corroborate those assumptions.

### 3.3. Qualitative Functional Characterization of the Silica Beads

#### 3.3.1. Infrared Spectroscopy

IR spectra of all studied silica nanoparticles (Figure S3) show the typical vibration bands with SiO_2_ at 793 cm^−1^ (Si–O–Si)_sym_, 945 cm^−1^ (Si-OH), 1060 cm^−1^ (Si–O–Si)_asym_ and 2930–3700 cm^−1^ for –OH stretching mode. With SiO_2_@NH_2_ and SiO_2_@PM vibrations were observed at 1495 cm^−1^ for CH_2_ and 2832 cm^−1^ for –CH stretching mode [74]. Very small vibrations corresponding the NH_2_ at 2930–3700 cm^−1^ for –NH stretching mode and 1485 cm^−1^ for NH_2_ bending mode were observed for SiO_2_@NH_2_ [75,76].

**Figure 4:**
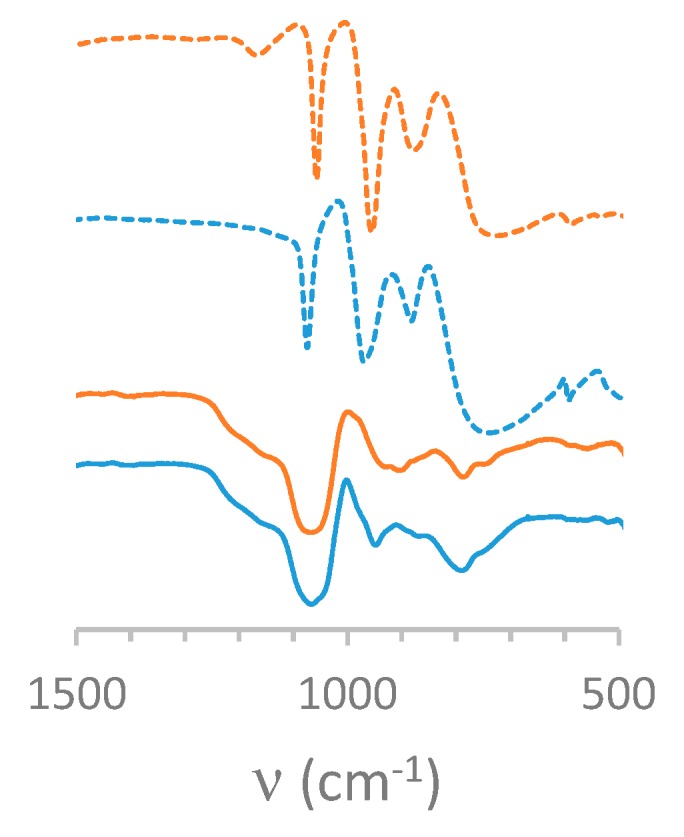
From up to down: Relevant IR vibration area for H_3_Pmo_12_O_40_, H_3_PW_12_O_40_ and difference spectra (SiO_2_@Pmo-SiO_2_@NH_2_) and (SiO_2_@PW-SiO_2_@NH_2_).

At the difference with mesoporous silica based materials [77], the content of APTES and PMs on non-porous silica beads was very low (only onto the surface of non-porous silica beads). Thus, it was not obvious to determine directly through IR if some APTES and PMs were grafted on the surface of SiO_2_ and SiO_2_@NH_2_ respectively. Some shouldering could be seen in SiO_2_@PMo and SiO_2_@PW at 950 cm^−1^ for P–O, 870 cm^−1^ for Mo=O and 805 cm^−1^ for Mo–O–Mo in case of SiO_2_@PMo, 1083–1092 cm^−1^ for P–O, 979–982 cm^−1^ for W=O, 897–901 cm^−1^ and 810–814 cm^−1^ for W–O–W in the case of SiO_2_@PW [66,78]. Those absorptions could correspond to the presence of polyanions. In addition, typical vibrations corresponding to Keggin units are overlapped with the ones of SiO_2_ [79]. An elegant method to prove the grafting was to do difference spectra between SiO_2_@NH_2_ and SiO_2_ (Figure S4) or SiO_2_@PM and SiO_2_@NH_2_ (Figure 4). Very small changes could be observed at 2926 and 1450–1700 cm^−1^ that could give a proof of the presence of the grafted APTES on SiO_2_ (Figure S4).

After addition of POMs, the difference spectra show vibrations corresponding to the POM backbone at 1060 cm^−1^ (P=O), 981 (W=O) and 873 cm^−1^ (W–O–W) for SiO_2_@PW or 944 (Mo=O) and 879 cm^−1^ (Mo–O–Mo) for SiO_2_@PMo with small shifts in comparison to pure POMs [53] (Figure 4). MAS NMR gave direct proofs of the grafting.

#### 3.3.2. Multinuclear Solid State NMR

Since IR did not give strongly affirmative answers concerning the nature of the functions surrounding the beads, solid state NMR has been an efficient tool. Indeed, ^1^H, ^13^C, ^29^Si and ^31^P are nuclei that can bring several information. All data have been summarized in Table S1. 

The ^1^H MAS NMR spectra exhibited very large signals attributed to different groups on silica beads, silanols and physisorbed water molecules (3.5–5 ppm), ethoxy (3.3–3.6 ppm) and methoxy (1.1–1.3 ppm) as well as the CH_2_ from the grafted silane (0.7–0.9 (Si-CH_2_), 6.5–6.8 (CH_2_-N) and 4.0–4.1 (CH_2_)) [80]. Unfortunately, large and/or overlapped signals were simply indicative. The ^13^C MAS NMR spectra show signals corresponding to the organic functions grafted on SiO_2_ and slight changes when POMs were added (see Table S1 and Figure S5) [81]. This method did confirm the silane and POM grafting.

**Figure 5:**
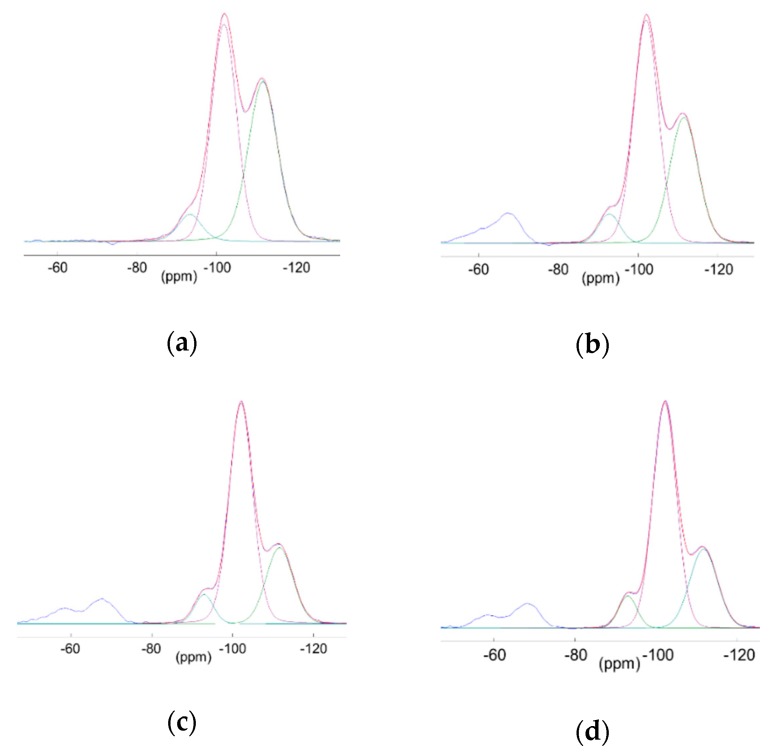
^29^Si CP MAS NMR spectra of SiO_2_ (**a**), SiO_2_@NH_2_ (**b**), SiO_2_@PW (**c**) and SiO_2_@PMo (**d**).

The ^29^Si CP MAS NMR (Table S1 and Figure 5) spectra gave additional information about the grafting on the silica bead itself. In all spectra, the signals at −93, −102 and −111 ppm corresponding to Q_2_, Q_3_ and Q_4_ respectively (Q_n_ = Si(Osi)_n_(OH)_4-n_) were in accordance with the SiO_2_ core [49]. The silane grafting was proved by two signals around −60 and −68 ppm (T_2_ and T_3_) [82]. A change of signals proportion was observed from SiO_2_ to SiO_2_@NH_2_ and from SiO_2_@NH_2_ to SiO_2_@PM, the trend being identical when both POMs were added. 

Since ^29^Si CP MAS NMR could not quantify the Q_n_, deconvolutions on SiO_2_ cores only were done on ^29^Si MAS NMR spectra, the relative intensity of the signals being indicated in parenthesis in Table S1. The stronger differences observed in ^29^Si CP-MAS (due to cross-polarization) were not so pronounced in ^29^Si MAS. (Figure S6) Those effects could be linked to the interactions between the ionic POMs and the SiO_2_@NH_2_ beads, once the proton exchange was performed.

Added POMs covering the SiO_2_@NH_2_ beads, different environments could be found, according to ionic interactions between charged POMs and pending ammonium, as well as H-interactions with silanol surfaces [83,84,85,86]. ^31^P MAS NMR signals of the grafted ones (Table S1) were shifted comparing to the value of the free POMs (the one used for the grafting) and relatively close to some referenced in the literature in ^31^P MAS for “PW_12_O_40_” [87] and “Pmo_12_O_40_” [88] backbones. 

### 3.4. Quantitative Functional Characterization of the Silica Beads

#### 3.4.1. Quantification by Elemental Analysis

**Scheme 2:**
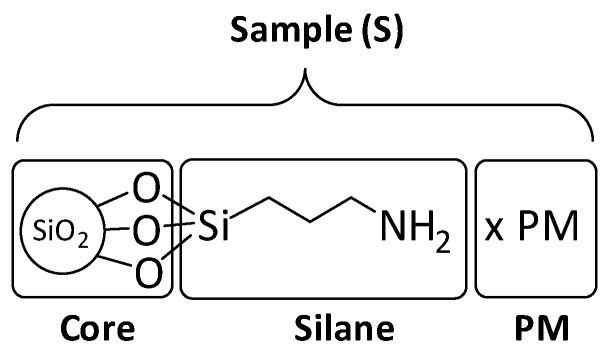
Schematic representation of the SiO_2_@NH_2_ and SiO_2_@PM beads.

From the nitrogen content (%N) found in elemental analysis, it is possible to calculate the number of moles of grafted aminosilane ρ(NH_2_) and POM grafted (ρ(PM)) per gram of sample S. The values could be compared to the one found using ^1^H liquid NMR. The number of moles of nitrogen atoms found in a sample S being equivalent to the number of NH_2_ fragments, the formula could be defined as follows in Equation (1). According to the Scheme 2, ρ(PM) could be calculated from the elemental analysis since we could postulate that the mixture of SiO_2_@NH_2_ with POM did a proton exchange from POM to the pending NH_2_ functions and no other modifications. ρ(NH_2_) and ρ(PM) are gathered in Table 1 and Table 2.

(1) $$\rho \left(NH_{2}\right)=\frac{n_{NH_{2}}}{m_{S}}=\frac{n_{N}}{m_{S}}=\frac{m_{N}}{m_{S}\cdot M_{N}}=\frac{\%N}{M_{N}}.$$

(2) $$\rho \left(PM\right)=\frac{n_{PM}}{m_{SiO_{2}@PM}}=x\cdot \rho \left(NH_{2}\right).$$

In order to find the x value, the important parameter is the mass of the SiO_2_ core within SiO_2_@NH_2_, obtained from %N values that corresponds to the number of grafted APTS fragments. This number is supposed to be unchanged after addition of POM, the variation observed in %N for SiO_2_@PMo and SiO_2_@PW will depend on the quantity of POMs retained by the beads (Equation (3))

(3) $$\%N_{SiO_{2}@PM}=\frac{M_{N}}{M_{SiO_{2}@NH_{2}}+x\cdot M_{PM}}.$$

M(SiO_2_@NH_2_) can be found using the N% of SiO_2_@NH_2_ (Equation (4))

(4) $$\%N_{SiO_{2}@NH_{2}}=\frac{M_{N}}{M_{SiO_{2}@NH_{2}}}.$$

Then, injecting in equation W, the x can be obtained from simple data using Equation (5).

(5) $$x=\frac{M_{N}}{M_{PM}}\left(\frac{1}{\%N_{SiO_{2}@PM}}-\frac{1}{\%N_{SiO_{2}@NH_{2}}}\right).$$

*x* will give the number of POM vs. N within the sample. According to the equation, the calculated data have been compilated in Table 1.

The ideal *x* value would have been 0.33, i.e., one POM retained by three NH_2_ functions. This could be due to the fact the some NH_2_ functions are “free” and the POM in the complete deprotonated form in the case of SiO_2_@PMo while in the case of SiO_2_@PW, the POM was not completely deprotonated.

#### 3.4.2. Quantification of Grafted Functions and Retained POM by Liquid NMR

Multinuclear liquid NMR (^1^H, and ^31^P) was used for this purpose with SiO_2_@NH_2_, SiO_2_@PMo and SiO_2_@PW beads. Silica beads and POMS can be destroyed in alkaline medium, giving silicates for the silica part, and tungstates/molybdates and phosphates for the POMs. It was proved that quantification could be done through the dissolution of the silica in aqueous solution in an alkali medium [89]. The organic backbone was maintained and could be quantified using an internal standard with ^1^H NMR. Using this method, the number of APTS fragments ρ(NH_2_) could be evaluated per gram of sample (Table 2).

Using the same methodology than the ^1^H NMR, the SiO_2_@PM was dissolved in very basic solution (pH = 13), in order to isolate the PO_4_^3−^. The ^31^P NMR signals obtained with the beads were quantified using an external calibration curve with different aqueous solutions of H_3_PO_4_ at pH = 13. The parameter found through this method (Table 2), ρ(PM), was relatively close to the one found through elemental analysis.

#### 3.4.3. Surface Coverage of the Beads Through EA and NMR.

Considering the number of functions present on one bead and the average size of the beads, using the classical density of SiO_2_, we could evaluate the surface coverage in number of functions per nm^2^. The demonstration of equation 6 is given in Supplementary information (Appendix A1).

(6) $$\mu \left(NH_{2}\right)=\frac{\rho^{\prime }\left(NH_{2}\right)\cdot r_{S}\cdot \rho_{SiO_{2}}}{3\times 10^{+21}}\times N_{A}.$$

Using those calculations, results (Table 2) indicated a relatively constant μ(NH_2_) value around 6.8 functions per nm^2^. This is in agreement with the hypothesis we assumed for the calculations on 3.4.1.

### 3.5. Catalysis

Homogenous catalysis with commercial POMs (especially H_3_PW_12_O_40_ and H_3_PMo_12_O_40_) has shown excellent activity in oxidation reactions [90,91,92,93,94,95,96,97]. Several examples have proven to be effective and the composition of the POMs can be modified for better selectivity [23,34]. In most of published experiments, organic solvent was needed, and the catalyst could not be recovered. Few examples described grafted POMs using Merrifield resins [34], polymeric quaternary ammonium salts [98], mesoporous supports [99,100,101], MOFs [102,103] or carbonaceous supports [104]. Mesoporous supports are interesting, but POMs entrapped within zeolites might be not all accessible. The advantage of the present process lies on complete accessibility of all POMs since only at the surface of the support. One drawback could be the lack of selectivity, but the reactions studied being quite simple, selectivity is not such a big issue. Following the grafting concept, SiO_2_@PM (M= Mo or W) was studied herein to achieve activity, recovery and reuse. A low ratio of POMs vs. substrate was tested. The SiO_2_@PM objects were recycled and used during three runs, with aqueous TBHP as an oxidant. The activity of the catalysts was tested on four model substrates. Cyclooctene (CO) is known to give essentially the cyclooctene oxide (COE) and few by-products. Cyclohexene (CH) gives cyclohexene oxide (CHO) and more products due to ring opening. Limonene (Lim) is a good biomass-issued substrate to study with different useful by-products. Cyclohexanol (CHol) is also interesting since its oxidation gives normally cyclohexanone, useful for the adipic acid synthesis. Relevant points are the low POM/substrate ratio used in the experiments (see tables) and no added organic solvent. This last point is something important towards the quest of chemical process tending to diminish the Green House effect [105].

#### 3.5.1. Cyclooctene (CO) Epoxidation.

**Scheme 3:**
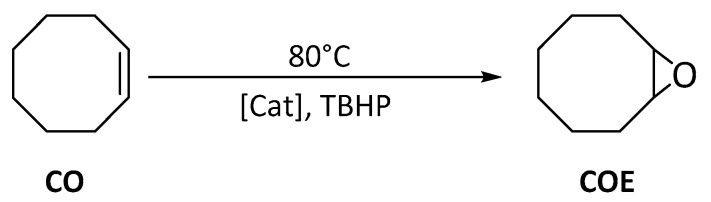
Catalyzed epoxidation of CO.

CO is interesting because the corresponding epoxide, cyclooctene oxide (COE) is known to be relatively stable towards ring-opening reactions. (Scheme 3) Although not frequent, hydrolysis and subsequent ring-opening might respectively lead to cyclooctanediol and suberic acid [106]. The oxidant (CO/TBHP ratio being 1:1.5) [23] is added once the temperature reached 80 °C. It must be pointed out that no organic solvent was added. The engaged mass of functionalized silica was identical but due to different POM/SiO_2_ grafting ratios between Mo and W experiments, the POM/substrate ratio differ. A relatively low POM/substrate ratio (0.070% and 0.058% for H_3_PW_12_O_40_ and H_3_PMo_12_O_40_ respectively) was added, among the lowest observed in the literature. Recovered catalysts were reused under the same experimental conditions to test the activity persistence. A test with non-grafted POMs was performed for comparison. Results have been compiled in Table 3.

**Figure 6:**
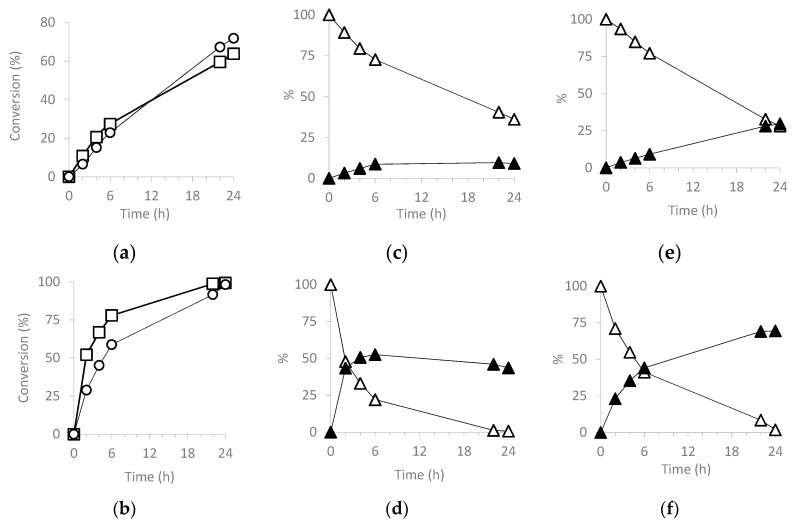
Comparison of CO conversion between (**a**) H_3_PW_12_O_40_ (□) and SiO_2_@PW (○), (**b**) H_3_PMo_12_O_40_ (□) and SiO_2_@PMo (○). Evolution of CO (△) and COE (▲) with (**c**) H_3_PW_12_O_40_, (**d**) H_3_PMo_12_O_40_, (**e**) SiO_2_@PW (Run 1) and (**f**) SiO_2_@PMo (Run 1).

Although the activity of SiO_2_@PM was slower during the first 6 h, CO conversions were almost the same than free POMs after 24 h but more selective towards COE in the case of the grafted POMs (Figure 6). The higher selectivity might be due to less acidic media with SiO_2_@PM. The SiO_2_@PMo catalyst was more active than SiO_2_@PW_12_, giving better CO conversion and higher selectivity towards COE. This trend was also observed with the free POMs. An interesting fact was the reuse of SiO_2_@PM. For both metals, catalytic performances were close during two extra runs (Figure S7; with average Turn Over Number (TON) values around 950 and 1640 for W and Mo respectively). 

The mechanism is not straightforward since the POMs are non-substituted and do not act as support of active metal as found in several other articles [107,108]. We suppose the formation of diperoxo on one metal, responsible to the oxygen transfer from perox to olefin [109].

#### 3.5.2. Cyclohexene (CH) (ep)oxidation

**Scheme 4:**
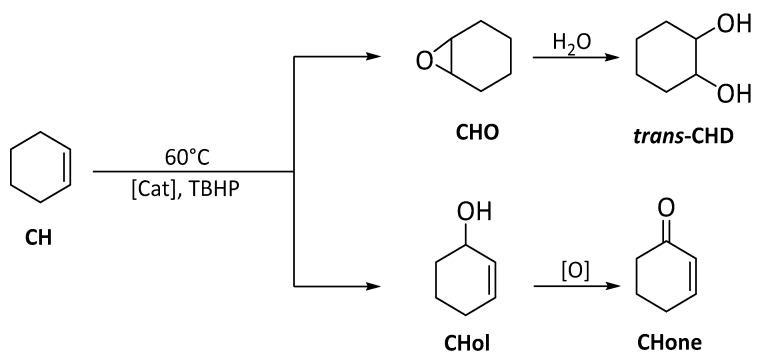
Oxidation reaction of CH.

(Ep)oxidation of CH, precursor of adipic acid [40] and simplified version of limonene, competes between epoxidation (CHO and the ring opening CHD) and allylic oxidation (CHol and CHone). The studies were done with the same TBHP ratio than for CO but with a five times lower catalyst charge than for CO (i.e., POM/CH ratio of 0.014% and 0.0116% for H_3_PW_12_O_40_ and H_3_PMo_12_O_40_ respectively) in order to exhibit the high activity of the catalysts. (Scheme 4) As we observed previously with Mo tridentate compounds [30], the epoxidation is the main reaction when TBHP was used as oxidant.

**Figure 7:**
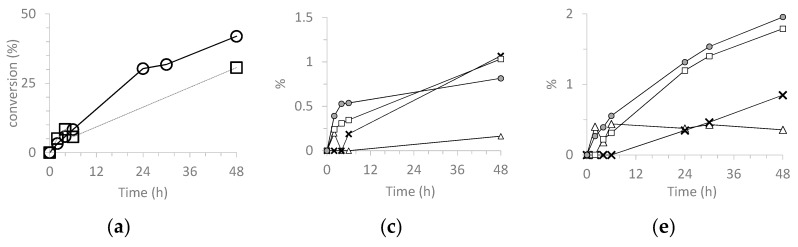

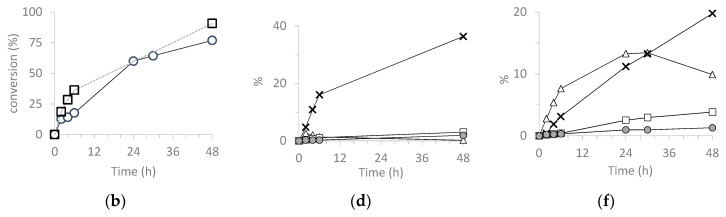
Comparison of conversion of CH between (**a**) H_3_PW_12_O_40_ (□) and SiO_2_@PW (○), (**b**) H_3_PMo_12_O_40_ (□) and SiO_2_@PMo (○). Evolution of CHO (△), CHD (×), CHol (□) and CHone (●) with (**c**) H_3_PW_12_O_40_, (**d**) H_3_PMo_12_O_40_, (**e**) SiO_2_@PW (Run 1) and (**f**) SiO_2_@PMo (Run 1).

After 48 h at 60 °C, as for CO substrate, SiO_2_@PMo was much more active than SiO_2_@PW_12_. (Table 4) The free H_3_PMo_12_O_40_ favored the epoxidation and the ring opening of the epoxide. The allylic oxidation seemed to be the preferred pathway with W containing species but not with a high difference towards Mo ones, showing that allylic oxidation did work without a catalyst (Figure 7). The reuse of the SiO_2_@PM exhibited interesting durability during two runs and the third started to show lower activity (Figure S8).

#### 3.5.3. Catalyzed Oxidation of Limonene

**Scheme 5:**
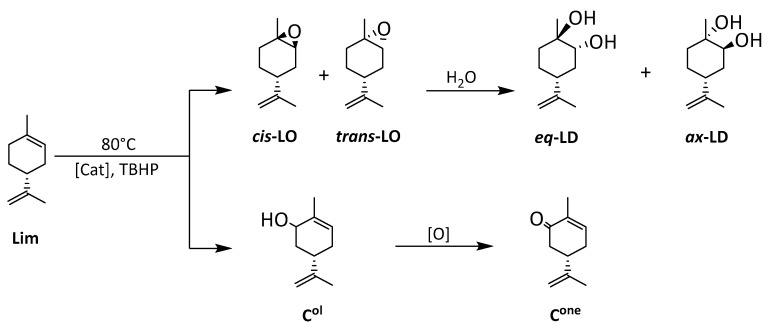
Oxidation reaction of limonene.

Oxidation reaction of limonene could lead to several different products corresponding to epoxidation with LO (*cis* and *trans*) and epoxide opening LD (*ax* and *eq*) and allylic oxidation (carveol C^ol^, and carvone C^one^) [27,29] (Scheme 5).

**Figure 8:**
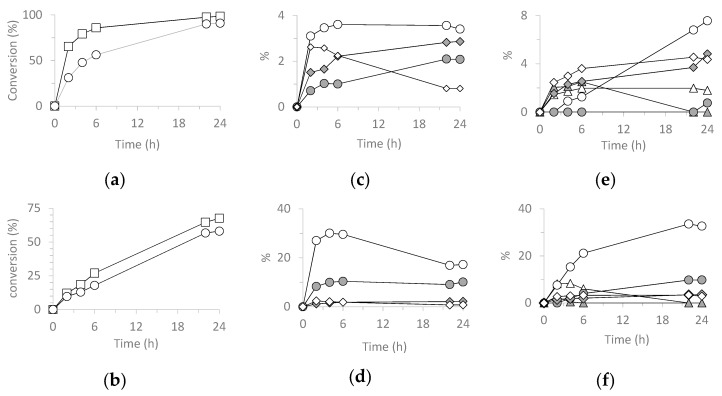
(**a**) Comparison of Lim conversion between (**a**) H_3_PW_12_O_40_ (□) and SiO_2_@PW (○), (**b**) H_3_PMo_12_O_40_ (□) and SiO_2_@PMo (○). Evolution of *trans*-LO (△), *cis*-LO CHD (▲), eq-LD (●), ax-LD (○), C^ol^ (◇) and C^one^ (◆) with (**c**) H_3_PW_12_O_40_, (**d**) H_3_PMo_12_O_40_, (**e**) SiO_2_@PW (Run 1) and (**f**) SiO_2_@PMo (Run 1).

Under the same conditions than for CO, results have been complied in Table 5. The results were strongly depending to the nature of the catalysts. The major products observed were *ax-* and *equ*-LD for SiO_2_@PMo, and carveol and carvone for SiO_2_@PW_12_ (Figure 8). This observation went in a straight line with observations done with CH.

Activity of SiO_2_@PMo was higher than SiO_2_@PW after each run (Figure S9). This could be also assumed by the absence of LO with Mo after 24 h, (but present at 6 h) and the presence of LDs. This was previously observed with other type of complexes [32]. Catalysts have a very weak influence on allylic oxidation, which could explain the similar selectivity of carveol and carvone. Durability of SiO_2_@PM was also proved by recycling with average TONs of 110 and 228 for SiO_2_@PW and SiO_2_@PMo respectively.

#### 3.5.4. Catalysis Oxidation of Cyclohexanol

**Scheme 6:**
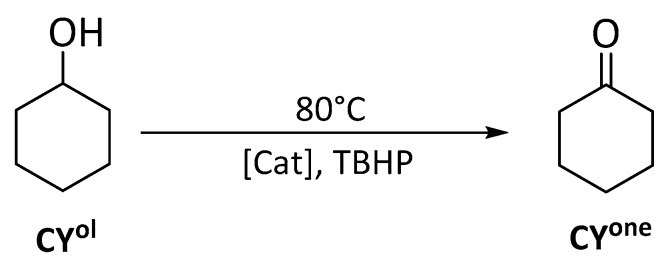
Catalyzed oxidation of CYol.

Cyclohexanol (CYol), as a precursor of adipid acid, i.e., one component of KA oil. Cyclohexanone (CYone) was the only oxidation product that was observed (Scheme 6). Under the same catalytic conditions than for CO and Lim, results have been compiled in Table 6.

**Figure 9:**
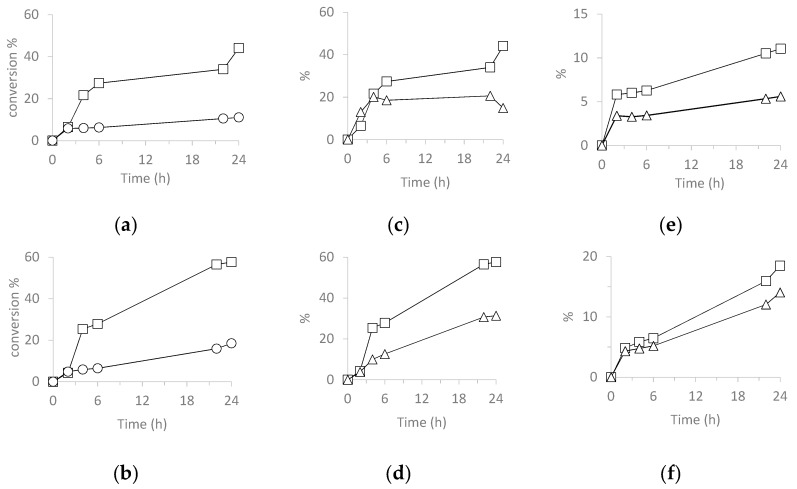
Comparison of CY^ol^ conversion between (**a**) H_3_PW_12_O_40_ (□) and SiO_2_@PW (○), (**b**) H_3_PMo_12_O_40_ (□) and SiO_2_@PMo (○). Conversion of CY^ol^ (□) and formation of CYone (△) with (**c**) H_3_PW_12_O_40_, (**d**) H_3_PMo_12_O_40_, (**e**) SiO_2_@PW (Run 1) and (**f**) SiO_2_@PMo (Run 1).

Both grafted catalysts had low conversion (Figure 9), SiO_2_@PMo being more active than SiO_2_@PW_12_ (with average conversion of 10% and 18% respectively) but with moderate activity compared to the free POMs. The mechanism might imply the formation of the diperoxo compound [110]. Although slow, the processes were more selective when grafted, certainly due to less acidic conditions. At the difference with other efficient processes using non-grafted compounds but microwave activation [111], recycling of the catalyst was possible (Figure S10) and interesting with similar conversions within the time. 

## 4. Conclusions

Ionic immobilization of POMs on silica beads functionalized by APTES led to the development of new catalytic materials (SiO_2_@PM) used for organic solvent-free (ep)oxidation reactions, showing with the studied substrates better selectivity than the corresponding free POMs. Morphological (DLS and TEM) studies of the SiO_2_@PM objects exhibited interesting behavior with ionic interactions going to dynamic particles agglomeration. This phenomenon seems to ensure the stability of non-monolithic recoverable catalytic objects, interesting in terms of potential industrial use. This methodology of catalysis uses organic solvent-free process, smoother oxidant, minimal catalyst loading and catalyst recyclability in a straight line with some principles of green chemistry. This environmentally benign protocol with a new type of catalytic materials can be easily modulated (other functionalization on beads, other grafted catalysts and other catalyzed reactions) and does open a new field of future investigations.

## Figures and Tables

**Table 1:** POM/NH_2_ molar ratio (x) obtained through elemental analysis (EA) data.

Sample	SiO_2_@NH_2_	SiO_2_@PW	SiO_2_@PMo
N (%)	0.74	0.43	0.58
x	0	0.47	0.29

**Table 2:** Calculated ρ(NH_2_) and ρ(PM) data and surface coverage μ(NH_2_).

Sample	ρ(NH_2_) ^1^ (^1^H NMR)	ρ(PM) ^1^ (^31^P NMR)	ρ(NH_2_) ^1^ (EA)	ρ(PM) ^1^ (EA)	μ(NH_2_) ^2^
**SiO_2_@NH_2_**	0.52	-	0.53	-	6.8
**SiO_2_@PW**	0.33	0.15	0.31	0.14	6.8
**SiO_2_@PMo**	0.40	0.12	0.41	0.12	6.7

^1^ in mmol functions/g sample, ^2^ in number functions/nm^2^.

**Table 3:** Relevant data for the catalyzed epoxidation of CO ^1^.

Catalyst	Run	CO Conv. ^2^	COE Sel. ^3^	TON ^4^
H_3_PW_12_O_40_	1	64	14	807
SiO_2_@PW	1	72	41	981
	2	75	38	987
	3	77	37	968
H_3_PMo_12_O_40_	1	99	44	1712
SiO_2_@PMo	1	98	71	1693
	2	96	72	1620
	3	93	69	1598

^1^ Conditions: 80 °C. POM/TBHP/CO = 0.070/150/100 for W, 0.058/150/100 for Mo; *t* = 24 h. ^2^ nCO converted/nCO engaged (in %) after 24 h. ^3^ nCOE formed/nCO converted (in %) at 24 h. ^4^ nCO transformed /nPOM at 24 h.

**Table 4:** Relevant data for the catalyzed (ep)oxidation of cyclohexene ^1^.

Catalyst	Run	CH Conv ^2^	CHO Sel ^3^	CHD Sel ^3^	Chol Sel ^3^	Chone Sel ^3^	TON ^4^
H_3_PW_12_O_40_	1	31	<1	4	3	3	11,307
SiO_2_@PW	1	45	1	2	4	5	21,458
	2	43	1	2	2	3	20,649
	3	26	3	3	7	7	12,373
H_3_PMo_12_O_40_	1	91	<1	40	3	2	52,728
SiO_2_@PMo	1	80	13	26	5	2	46,732
	2	74	15	22	6	3	42,487
	3	60	26	20	4.9	2	36,345

^1^ Conditions: 80 °C. POM/TBHP/CH = 0.014/150/100 for W, 0.0116/150/100 for Mo; *t* = 48 h. ^2^ nCH converted/nCH engaged (%) after 48 h. ^3^ n product formed/nCH converted (%) at 48 h. ^4^ nCH transformed /nPOM at 48 h.

**Table 5:** Relevant data for the catalyzed oxidation of limonene ^1^.

Catalyst	Run	Lim Conv ^2^	*cis*-LO Sel ^3^	*trans*-LO Sel ^3^	*ax*-LD Sel ^3^	*eq*-LD Sel ^3^	C^ol^ Sel ^3^	C^one^ Sel ^3^	TON ^4^
H_3_PW_12_O_40_	1	67	0	0	5	3	1	4	1287
SiO_2_@PW	1	58	0	3	13	1	8	8	754
	2	59	0	3	13	1	10	8	768
	3	62	0	2	12	2	11	8	754
H_3_PMo_12_O_40_	1	99	0	0	18	10	1	2	1859
SiO_2_@PMo	1	91	0	0	36	11	4	3	1721
	2	86	0	0	32	8	6	7	1626
	3	81	0	< 1	20	5	6	6	1526

^1^ Conditions: 80 °C. POM/TBHP/Lim=0.070/150/100 for W, 0.058/150/100 for Mo; *t* = 24 h. ^2^ nLim converted/nLim engaged (in%) after 24 h. ^3^ n product formed/nLim converted at 24 h. ^4^ nLim transformed /nPOM at 24 h.

**Table 6:** Relevant data for the catalyzed oxidation of cyclohexanol after 24 h ^1.^

Catalyst	Run	CY^ol^ Conversion ^2^	CY^one^ Selectivity ^3^	TON ^4^
H_3_PW_12_O_40_	1	44	34	525
SiO_2_@PW	1	11	51	137
	2	8	97	101
	3	7	87	92
H_3_PMo_12_O_40_	1	58	54	728
SiO_2_@PMo	1	18	76	228
	2	17	90	207
	3	20	75	249

^1^ Conditions: 80 °C. POM/TBHP/CYol=0.070/150/100 for W, 0.058/150/100 for Mo; *t* = 24 h. ^2^ n CYol converted/n CYol engaged (in %) after 24 h. ^3^ n CYone formed/nCYol converted (in %) at 24 h. ^4^ n CYol transformed /nPOM at 24 h.

## Supplementary Materials

The following are available online at https://www.mdpi.com/1996-1944/12/20/3278/s1, **Figure S1**: Powder X-ray diffraction diagrams of **SiO_2_** (blue), **SiO_2_@PW** (orange) and **SiO_2_@PMo** (grey) particles. **Figure S2**: Comparison of Powder X-ray diffractions of (a) H_3_PMo_12_O_40_ (orange) and **SiO_2_@PMo** (blue) and (b) H_3_PW_12_O_40_ (orange) and **SiO_2_@PW** (blue). The intensities of **SiO_2_@PMo** and **SiO_2_@PW** were magnified 10 times. **Figure S3**: From up to down: Relevant IR vibration zones for **SiO_2_**, **SiO_2_@NH_2_**, **SiO_2_@PW**, **SiO_2_@PMo**. **Figure S4**: Difference spectra (**SiO_2_@NH_2_** - **SiO_2_**) on specific ranges (in blue). The spectrum of APTES is indicated in orange **Table S1**: Relevant solid-state NMR data. **Figure S5**: ^13^C MAS NMR spectra of **SiO_2_@PW** (up), **SiO_2_@PMo** (middle) and **SiO_2_@NH_2_** (down). **Figure S6**: ^29^Si MAS NMR spectra of SiO_2_ (a) SiO_2_@NH_2_ (b), SiO_2_@PW (c) and SiO_2_@PMo (d). Appendix A1- Determination function coverage of functionalized silica beads. **Figure S7**: Evolution of CO (△) and COE (▲) with **SiO_2_@PW** (Run 1 (a), Run 2 (b) and Run 3 (c)) and **SiO_2_@PMo** (Run 1 (d), Run 2 (e) and Run 3 (f)). **Figure S8**: Evolution of CHO (△), CHD (×), CHol (□) and CHone (●) with **SiO_2_@PW** (Run 1 (a), Run 2 (b) and Run 3 (c)) and **SiO_2_@PMo** (Run 1 (d), Run 2 (e) and Run 3 (f)). **Figure S9**: (a) Evolution of *trans*-LO (△), *cis*-LO CHD (▲), eq-LD (●), ax-LD (○), C^ol^ (◇) and C^one^ (◆) with **SiO_2_@PW** (Run 1 (a), Run 2 (b) and Run 3 (c)) and **SiO_2_@PMo** (Run 1 (d), Run 2 (e) and Run 3 (f)). **Figure S10**: Conversion of CY^ol^ (□) and formation of CYone (△) with **SiO_2_@PW** (Run 1 (a), Run 2 (b) and Run 3 (c)) and **SiO_2_@PMo** (Run 1 (d), Run 2 (e) and Run 3 (f)).

## References

[1] Pastor I.M., Yus M. (2005). Asymmetric Ring Opening of Epoxides. Curr. Org. Chem..

[2] Matlock P.L., Brown W.L., Clinton N.A. (1999). Polyalkylene glycols. Chem. Ind..

[3] Petrović Z.S. (2008). Polyurethanes from Vegetable Oils. Polym. Rev..

[4] McCann S.D., Stahl S.S. (2015). Copper-Catalyzed Aerobic Oxidations of Organic Molecules: Pathways for Two-Electron Oxidation with a Four-Electron Oxidant and a One-Electron Redox-Active Catalyst. Acc. Chem. Res..

[5] Bowman S., Ide M.S., Davis R.J. (2013). Selective Oxidation of Alcohols and Aldehydes over Supported Metal Nanoparticles. Green Chem..

[6] Ciriminna R., Pandarus V., Béland F., Xu Y.-J., Pagliaro M. (2015). Heterogeneously Catalyzed Alcohol Oxidation for the Fine Chemical Industry. Org. Process. Res. Dev..

[7] Parmeggiani C., Matassini C., Cardona F. (2017). A Step Forward towards Sustainable Aerobic Alcohol Oxidation: New and Revised Catalysts Based on Transition Metals on Solid Supports. Green Chem..

[8] Sheldon R.A. (2015). Recent Advances in Green Catalytic Oxidations of Alcohols in Aqueous Media. Catal. Today.

[9] Marais L., Swarts A.J. (2019). Biomimetic Cu/Nitroxyl Catalyst Systems for Selective Alcohol Oxidation. Catalysts.

[10] Bai X., Huang X., Wen L., Song N., Zhang J., Zhang Y., Zhao Y. (2019). A New Strategy for the Selective Oxidation of Alcohols Catalyzed by a Polyoxometalate-Based Hybrid Surfactant in Biphasic Systems. Chem. Commun..

[11] McDonald R.N., Steppel R.N., Dorsey J.E. (1970). m-Chloroperbenzoic acid. Org. Synth..

[12] Rose E., Andrioletti B., Zrig S., Quelquejeu-Ethève M. (2005). Enantioselective Epoxidation of Olefins with Chiral Metalloporphyrin Catalysts. Chem. Soc. Rev..

[13] Swern D. (1971). Organic Peroxides.

[14] Plesnicar B., Trahanovsky W.C. (1978). Organic Chemistry, Part C.

[15] Kobayashi S., Tawara K. Method for producing epoxy compound. Patent.

[16] Sherwood J. (2018). European Restrictions on 1,2-Dichloroethane: C−H Activation Research and Development Should Be Liberated and Not Limited. Angew. Chem. Int. Ed..

[17] Sheldon R.A., Arends I., Hanefeld U. (2007). Green Chemistry and Catalysis.

[18] Anastas P., Warner J. (1998). Green Chemistry: Theory and Practice.

[19] Tundo P., Anastas P., Black D.S., Breen J., Collins T.J., Memoli S., Miyamoto J., Polyakoff M., Tumas W. (2000). Synthetic Pathways and Processes in Green Chemistry. Introductory Overview. Pure Appl. Chem..

[20] Pisk J., Agustin D., Vrdoljak V., Poli R. (2011). Epoxidation Processes by Pyridoxal Dioxomolybdenum(VI) (Pre)Catalysts Without Organic Solvent. Adv. Synth. Catal..

[21] Pisk J., Prugovečki B., Matković-Čalogović D., Poli R., Agustin D., Vrdoljak V. (2012). Charged Dioxomolybdenum(VI) Complexes with Pyridoxal Thiosemicarbazone Ligands as Molybdenum(V) Precursors in Oxygen Atom Transfer Process and Epoxidation (Pre)Catalysts. Polyhedron.

[22] Morlot J., Uyttebroeck N., Agustin D., Poli R. (2013). Solvent-Free Epoxidation of Olefins Catalyzed by “[MoO_2_(SAP)]”: A New Mode of Tert-Butylhydroperoxide Activation. ChemCatChem.

[23] Guérin B., Mesquita Fernandes D., Daran J.-C., Agustin D., Poli R. (2013). Investigation of Induction Times, Activity, Selectivity, Interface and Mass Transport in Solvent-Free Epoxidation by H_2_O_2_ and TBHP: A Study with Organic Salts of the [PMo_12_O_40_]^3−^ Anion. New J. Chem..

[24] Wang W., Vanderbeeken T., Agustin D., Poli R. (2015). Tridentate ONS vs. ONO Salicylideneamino(Thio)Phenolato [MoO_2_L] Complexes for Catalytic Solvent-Free Epoxidation with Aqueous TBHP. Catal. Commun..

[25] Vrdoljak V., Pisk J., Agustin D., Novak P., Vuković J.P., Matković-Čalogović D. (2014). Dioxomolybdenum(VI) and Dioxotungsten(VI) Complexes Chelated with the ONO Tridentate Hydrazone Ligand: Synthesis, Structure and Catalytic Epoxidation Activity. New J. Chem..

[26] Wang W., Daran J.-C., Poli R., Agustin D. (2016). OH-Substituted Tridentate ONO Schiff Base Ligands and Related Molybdenum(VI) Complexes for Solvent-Free (Ep)Oxidation Catalysis with TBHP as Oxidant. J. Mol. Cat. A Chem..

[27] Wang W., Agustin D., Poli R. (2017). Influence of Ligand Substitution on Molybdenum Catalysts with Tridentate Schiff Base Ligands for the Organic Solvent-Free Oxidation of Limonene Using Aqueous TBHP as Oxidant. Mol. Catal..

[28] Pisk J., Rubčić M., Kuzman D., Cindrić M., Agustin D., Vrdoljak V. (2019). Molybdenum(VI) Complexes of Hemilabile Aroylhydrazone Ligands as Efficient Catalysts for Greener Cyclooctene Epoxidation: An Experimental and Theoretical Approach. New J. Chem..

[29] Cindrić M., Pavlović G., Katava R., Agustin D. (2017). Towards a Global Greener Process: From Solvent-Less Synthesis of Molybdenum(VI) ONO Schiff Base Complexes to Catalyzed Olefin Epoxidation under Organic-Solvent-Free Conditions. New J. Chem..

[30] Pisk J., Daran J.-C., Poli R., Agustin D. (2015). Pyridoxal Based ONS and ONO Vanadium(V) Complexes: Structural Analysis and Catalytic Application in Organic Solvent Free Epoxidation. J. Mol. Cat. A Chem..

[31] Damjanović V., Pisk J., Kuzman D., Agustin D., Vrdoljak V., Stilinović V., Cindrić M. (2019). The Synthesis, Structure and Catalytic Properties of the [Mo_7_O_24_(μ-Mo_8_O_26_)Mo_7_O_24_]^16−^ Anion Formed via Two Intermediate Heptamolybdates [Co(en)_3_]_2_[NaMo_7_O_24_]Cl·nH_2_O and (H_3_O)[Co(En)_3_]_2_[Mo_7_O_24_]Cl·9H_2_O. Dalton Trans..

[32] Landau R., Sullivan G.A., Brown D. (1979). Propylene Oxide by the Co-Product Processes. Chemtech.

[33] Sutradhar M., Martins L.M., Da Silva M.F.C.G., Pombeiro A.J. (2015). Vanadium complexes: Recent progress in oxidation catalysis. Coord. Chem. Rev..

[34] Pisk J., Agustin D., Poli R. (2019). Organic Salts and Merrifield Resin Supported [PM_12_O_40_]^3−^ (M = Mo or W) as Catalysts for Adipic Acid Synthesis. Molecules.

[35] Maurya M.R. (2018). Vanadium Complexes Based Polymer Supported Catalysts: A Brief Account of Research from Our Group. Top. Catal..

[36] Sutradhar M., Martins L.M.D.R.S., Carabineiro S.A.C., Da Silva M.F.C.G., Buijnsters J.G., Figueiredo J.L., Pombeiro A.J.L. (2016). Oxidovanadium(V) Complexes Anchored on Carbon Materials as Catalysts for the Oxidation of 1-Phenylethanol. ChemCatChem.

[37] Richards R., Kortz U., Bi L., Zhu K., Suchopar A., Cheng L. Supported Polyoxometallates and Process for Their Preparation. Patent.

[38] Balula S.S., Cunha-Silva L., Santos I.C.M.S., Estrada A.C., Fernandes A.C., Cavaleiro J.A.S., Pires J., Freire C., Cavaleiro A.M.V. (2013). Mono-Substituted Silicotungstates as Active Catalysts for Sustainable Oxidations: Homo- and Heterogeneous Performance. New J. Chem..

[39] Panchenko V.N., Borbáth I., Timofeeva M.N., Gőbölös S. (2010). Amine-Modified Silica NH_2_–(CH_2_)x–SiO_2_ (X=0, 2, 3) as Support for Cobalt-Substituted Polyoxometalate TBA_4_HPW_11_CoO_39_: Effect of the Nature of the Support on the Oxidation Activity. J. Mol. Cat. A Chem..

[40] Li X., Jiang P., Wang Z., Huang Y. (2016). Phosphotungstate-Based Ionic Silica Nanoparticles Network for Alkenes Epoxidation. Catalysts.

[41] Neumann R., Miller H. (1995). Alkene Oxidation in Water Using Hydrophobic Silica Particles Derivatized with Polyoxometalates as Catalysts. J. Chem. Soc. Chem. Commun..

[42] Simon A., Cohen-Bouhacina T., Porté M.C., Aimé J.P., Baquey C. (2002). Study of Two Grafting Methods for Obtaining a 3-Aminopropyltriethoxysilane Monolayer on Silica Surface. J. Colloid Interface Sci..

[43] Cuoq F., Masion A., Labille J., Rose J., Ziarelli F., Prelot B., Bottero J.-Y. (2013). Preparation of Amino-Functionalized Silica in Aqueous Conditions. Appl. Surf. Sci..

[44] Qiao B., Wang T.-J., Gao H., Jin Y. (2015). High Density Silanization of Nano-Silica Particles Using γ-Aminopropyltriethoxysilane (APTES). Appl. Surf. Sci..

[45] Okun N.M., Anderson T.M., Hill C.L. (2003). [(Fe^III^(OH_2_)_2_)_3_(A-α-PW_9_O_34_)_2_]^9−^ on Cationic Silica Nanoparticles, a New Type of Material and Efficient Heterogeneous Catalyst for Aerobic Oxidations. J. Am. Chem. Soc..

[46] Okun N.M., Anderson T.M., Hill C.L. (2003). Polyoxometalates on Cationic Silica: Highly Selective and Efficient O_2_/Air-Based Oxidation of 2-Chloroethyl Ethyl Sulfide at Ambient Temperature. J. Mol. Cat. A Chem..

[47] Wu W.-J., Wang J., Chen M., Qian D.-J., Liu M. (2017). Terpyridine-Functionalized NanoSiO_2_ Multi-Dentate Linkers: Preparation, Characterization and Luminescent Properties of Their Metal–Organic Hybrid Materials. J. Phys. Chem. C.

[48] Wang J., Hao Q. (2009). Thin Films of Silica Particles Covered with Lanthanide Substituted Keggin Polyoxometalates and Their Optical Properties. J. Alloys Compd..

[49] Sharma R.K., Sharma S., Gulati S., Pandey A. (2013). Fabrication of a Novel Nano-Composite Carbon Paste Sensor Based on Silica-Nanospheres Functionalized with Isatin Thiosemicarbazone for Potentiometric Monitoring of Cu^2+^ Ions in Real Samples. Anal. Methods.

[50] Lee J.H., Kim J.H., Choi K., Kim H.G., Park J.A., Cho S.H., Hong S.W., Lee J., Lee J.H., Lee S.-J. (2018). Investigation of the Mechanism of Chromium Removal in (3-Aminopropyl)Trimethoxysilane Functionalized Mesoporous Silica. Sci. Rep..

[51] Goscianska J., Olejnik A., Nowak I. (2017). APTES-Functionalized Mesoporous Silica as a Vehicle for Antipyrine – Adsorption and Release Studies. Colloids Surf. A.

[52] Nogueira L.S., Ribeiro S., Granadeiro C.M., Pereira E., Feio G., Cunha-Silva L., Balula S.S. (2014). Novel Polyoxometalate Silica Nano-Sized Spheres: Efficient Catalysts for Olefin Oxidation and the Deep Desulfurization Process. Dalton Trans..

[53] Okun N.M., Ritorto M.D., Anderson T.M., Apkarian R.P., Hill C.L. (2004). Polyoxometalates on Cationic Silica Nanoparticles. Physicochemical Properties of an Electrostatically Bound Multi-Iron Catalyst. Chem. Mater..

[54] Naz A., Arun S., Narvi S.S., Alam M.S., Singh A., Bhartiya P., Dutta P.K. (2018). Cu(II)-Carboxymethyl Chitosan-Silane Schiff Base Complex Grafted on Nano Silica: Structural Evolution, Antibacterial Performance and Dye Degradation Ability. Int. J. Biol. Macromol..

[55] Talreja K., Chauhan I., Ghosh A., Majumdar A., Butola B.S. (2017). Functionalization of Silica Particles to Tune the Impact Resistance of Shear Thickening Fluid Treated Aramid Fabrics. RSC Adv..

[56] Van den Berg R., Parmentier T.E., Elkjær C.F., Gommes C.J., Sehested J., Helveg S., de Jongh P.E., de Jong K.P. (2015). Support Functionalization To Retard Ostwald Ripening in Copper Methanol Synthesis Catalysts. ACS Catal..

[57] Sándor M., Nistor C.L., Szalontai G., Stoica R., Nicolae C.A., Alexandrescu E., Fazakas J., Oancea F., Donescu D. (2016). Aminopropyl-Silica Hybrid Particles as Supports for Humic Acids Immobilization. Materials.

[58] Blair M., Andrews P.C., Fraser B.H., Forsyth C.M., Junk P.C., Massi M., Tuck K.L. (2007). Facile Methods for the Separation of the Cis- and Trans-Diastereomers of Limonene 1,2-Oxide and Convenient Routes to Diequatorial and Diaxial 1,2-Diols. Synthesis.

[59] Van der Werf M.J., Jongejan H., Franssen M.C.R. (2001). Resolution of Limonene 1,2-Epoxide Diastereomers by Mercury(II) Ions. Tetrahedron Lett..

[60] Steiner D., Ivison L., Goralski C.T., Appell R.B., Gojkovic J.R., Singaram B. (2002). A Facile and Efficient Method for the Kinetic Separation of Commercially Available Cis- and Trans-Limonene Epoxide. Tetrahedron Asymmetry.

[61] Stöber W., Fink A., Bohn E. (1968). Controlled Growth of Monodisperse Silica Spheres in the Micron Size Range. J. Colloid Interface Sci..

[62] Bu J., Li R., Quah C.W., Carpenter K.J. (2004). Propagation of PAMAM Dendrons on Silica Gel: A Study on the Reaction Kinetics. Macromolecules.

[63] Dolbecq A., Dumas E., Mayer C.R., Mialane P. (2010). Hybrid Organic–Inorganic Polyoxometalate Compounds: From Structural Diversity to Applications. Chem. Rev..

[64] Azarshin S., Moghadasi J., A Aboosadi Z. (2017). Surface Functionalization of Silica Nanoparticles to Improve the Performance of Water Flooding in Oil Wet Reservoirs. Energy Explor. Exploit..

[65] Gholami T., Salavati-Niasari M., Bazarganipour M., Noori E. (2013). Synthesis and Characterization of Spherical Silica Nanoparticles by Modified Stöber Process Assisted by Organic Ligand. Superlattices Microstruct..

[66] Cai W., Zhou Y., Bao R., Yue B., He H. (2013). Catalytic Epoxidation of Cyclohexene over Mesoporous-Silica Immobilized Keggin-Type Tungstophosphoric Acid. Chin. J. Catal..

[67] Stetefeld J., McKenna S.A., Patel T.R. (2016). Dynamic Light Scattering: A Practical Guide and Applications in Biomedical Sciences. Biophys. Rev..

[68] Kobayashi M., Juillerat F., Galletto P., Bowen P., Borkovec M. (2005). Aggregation and Charging of Colloidal Silica Particles: Effect of Particle Size. Langmuir.

[69] Mansfield E.D., Pandya Y., Mun E.A., Rogers S.E., Abutbul-Ionita I., Danino D., Williams A.C., Khutoryanskiy V.V. (2018). Structure and Characterisation of Hydroxyethylcellulose–Silica Nanoparticles. RSC Adv..

[70] Neves C.S., Granadeiro C.M., Cunha-Silva L., Ananias D., Gago S., Feio G., Carvalho P.A., Eaton P., Balula S.S., Pereira E. (2013). Europium Polyoxometalates Encapsulated in Silica Nanoparticles—Characterization and Photoluminescence Studies. Eur. J. Inorg. Chem..

[71] Greasley S.L., Page S.J., Sirovica S., Chen S., Martin R.A., Riveiro A., Hanna J.V., Porter A.E., Jones J.R. (2016). Controlling Particle Size in the Stöber Process and Incorporation of Calcium. J. Colloid Interface Sci..

[72] Mahalingam V., Onclin S., Péter M., Ravoo B.J., Huskens J., Reinhoudt D.N. (2004). Directed Self-Assembly of Functionalized Silica Nanoparticles on Molecular Printboards through Multivalent Supramolecular Interactions. Langmuir.

[73] Martín S., Takashima Y., Lin C.-G., Song Y.-F., Miras H.N., Cronin L. (2019). Integrated Synthesis of Gold Nanoparticles Coated with Polyoxometalate Clusters. Inorg. Chem..

[74] Aneja K.S., Bohm S., Khanna A.S., Mallika Bohm H.L. (2015). Graphene Based Anticorrosive Coatings for Cr(VI) Replacement. Nanoscale.

[75] Karimpour T., Safaei E., Karimi B., Lee Y.-I. (2018). Iron(III) Amine Bis(Phenolate) Complex Immobilized on Silica-Coated Magnetic Nanoparticles: A Highly Efficient Catalyst for the Oxidation of Alcohols and Sulfides. ChemCatChem.

[76] Tighadouini S., Radi S., Elidrissi A., Zaghrioui M., Garcia Y. (2019). Selective Confinement of CdII in Silica Particles Functionalized with β-Keto-Enol-Bisfuran Receptor: Isotherms, Kinetic and Thermodynamic Studies. Eur. J. Inorg. Chem..

[77] Luo X., Yang C. (2011). Photochromic Ordered Mesoporous Hybrid Materials Based on Covalently Grafted Polyoxometalates. Phys. Chem. Chem. Phys..

[78] Kumar D., Landry C.C. (2007). Immobilization of a Mo, V-Polyoxometalate on Cationically Modified Mesoporous Silica: Synthesis and Characterization Studies. Microporous Mesoporous Mater..

[79] Misono M., Mizuno N., Katamura K., Kasai A., Konishi Y., Sakata K., Okuhara T., Yoneda Y. (1982). Catalysis by Heteropoly Compounds. III. The Structure and Properties of 12-Heteropolyacids of Molybdenum and Tungsten (H_3_PMo_12_−xWxO_40_) and Their Salts Pertinent to Heterogeneous Catalysis. Bull. Chem. Soc. Jpn..

[80] Trébosc J., Wiench J.W., Huh S., Lin V.S.-Y., Pruski M. (2005). Solid-State NMR Study of MCM-41-Type Mesoporous Silica Nanoparticles. J. Am. Chem. Soc..

[81] Hicks J.C., Jones C.W. (2006). Controlling the Density of Amine Sites on Silica Surfaces Using Benzyl Spacers. Langmuir.

[82] Ribeiro S.O., Granadeiro C.M., Almeida P.L., Pires J., Capel-Sanchez M.C., Campos-Martin J.M., Gago S., de Castro B., Balula S.S. (2019). Oxidative Desulfurization Strategies Using Keggin-Type Polyoxometalate Catalysts: Biphasic versus Solvent-Free Systems. Catal. Today.

[83] Bentaleb F., Makrygenni O., Brouri D., Coelho Diogo C., Mehdi A., Proust A., Launay F., Villanneau R. (2015). Efficiency of Polyoxometalate-Based Mesoporous Hybrids as Covalently Anchored Catalysts. Inorg. Chem..

[84] Cheng C.-Y., Lin K.-J., Prasad M.R., Fu S.-J., Chang S.-Y., Shyu S.-G., Sheu H.-S., Chen C.-H., Chuang C.-H., Lin M.-T. (2007). Synthesis of a Reusable Oxotungsten-Containing SBA-15 Mesoporous Catalyst for the Organic Solvent-Free Conversion of Cyclohexene to Adipic Acid. Catal. Commun..

[85] Xiao Y., Chen D., Ma N., Hou Z., Hu M., Wang C., Wang W. (2013). Covalent Immobilization of a Polyoxometalate in a Porous Polymer Matrix: A Heterogeneous Catalyst towards Sustainability. RSC Adv..

[86] Mizuno N., Yamaguchi K., Kamata K. (2011). Molecular Design of Polyoxometalate-Based Compounds for Environmentally-Friendly Functional Group Transformations: From Molecular Catalysts to Heterogeneous Catalysts. Catal. Surv. Asia.

[87] Kozhevnikov I.V., Kloetstra K.R., Sinnema A., Zandbergen H.W., van Bekkum H. (1996). Study of Catalysts Comprising Heteropoly Acid H_3_PW_12_O_40_ Supported on MCM-41 Molecular Sieve and Amorphous Silica. J. Mol. Catal. A Chem..

[88] Canioni R., Roch-Marchal C., Sécheresse F., Horcajada P., Serre C., Hardi-Dan M., Férey G., Grenèche J.-M., Lefebvre F., Chang J.-S. (2011). Stable Polyoxometalate Insertion within the Mesoporous Metal Organic Framework MIL-100(Fe). J. Mater. Chem..

[89] Crucho C.I.C., Baleizão C., Farinha J.P.S. (2017). Functional Group Coverage and Conversion Quantification in Nanostructured Silica by ^1^H NMR. Anal. Chem..

[90] Nlate S., Kortz U., Bonchio M. (2012). Polyoxometalate as Homogeneous Oxidation Catalysts. Innovative Catalysis in Organic Synthesis: Oxidation, Hydrogenation, and C-X Bond Forming Reaction.

[91] Neumann R., Borrás-Almenar J.J., Coronado E., Müller A., Pope M. (2003). Applications of Polyoxometalates in Homogeneous Catalysis. Polyoxometalate Molecular Science.

[92] Rafiee E., Eavani S. (2017). Polyoxometalates as Heterogeneous Catalysts for Organic Reactions. Curr. Org. Chem..

[93] Duprez D., Cavani F. (2014). Handbook of Advanced Methods and Processes in Oxidation Catalysis: From Laboratory to Industry.

[94] Carraro M., Fiorani G., Sartorel A., Bonchio M. (2011). Polyoxometalates Catalysts for Sustainable Oxidations and Energy Applications. Handbook of Advanced Methods and Processes in Oxidation Catalysis.

[95] Kholdeeva O.A., Maksimchuk N.V., Maksimov G.M. (2010). Polyoxometalate-Based Heterogeneous Catalysts for Liquid Phase Selective Oxidations: Comparison of Different Strategies. Catal. Today.

[96] Ma X., He P., Xu B., Lu J., Wan R., Wu H., Wang Y., Ma P., Niu J., Wang J. (2019). Pyrazine dicarboxylate-bridged arsenotungstate: Synthesis, characterization, and catalytic activities in epoxidation of olefins and oxidation of alcohols. Dalton Trans..

[97] Zhang T.-T., Zhang X., Lü Y., Li G.-D., Xiao L.-N., Cui X.-B., Xu J.-Q. (2017). New organic–inorganic hybrid compounds based on [SiNb_12_V_2_O_42_]^12−^ with high catalytic activity for styrene epoxidation. Inorg. Chem. Front..

[98] Zhang L., Song S., Yang N., Tantai X., Xiao X., Jiang B., Sun Y. (2019). Porous Hybrid Nanoflower Self-Assembled from Polyoxometalate and Polyionene for Efficient Oxidative Desulfurization. Ind. Eng. Chem. Res..

[99] Masteri-Farahani M., Modarres M. (2016). Wells-Dawson Heteropoly Acid Immobilized inside the Nanocages of SBA-16 with Ship-in-a-Bottle Method: A New Recoverable Catalyst for the Epoxidation of Olefins. J. Mol. Catal. A Chem..

[100] Song X., Zhu W., Li K., Wang J., Niu H., Gao H., Gao W., Zhang W., Yu J., Jia M. (2016). Epoxidation of Olefins with Oxygen/Isobutyraldehyde over Transition-Metal-Substituted Phosphomolybdic Acid on SBA-15. Catal. Today.

[101] Karimi Z., Mahjoub A.R., Harati S.M. (2011). Polyoxometalate-Based Hybrid Mesostructured Catalysts for Green Epoxidation of Olefins. Inorg. Chim. Acta.

[102] Gao W., Sun X., Niu H., Song X., Li K., Gao H., Zhang W., Yu J., Jia M. (2015). Phosphomolybdic Acid Functionalized Covalent Organic Frameworks: Structure Characterization and Catalytic Properties in Olefin Epoxidation. Microporous Mesoporous Mater..

[103] Song X., Hu D., Yang X., Zhang H., Zhang W., Li J., Jia M., Yu J. (2019). Polyoxomolybdic Cobalt Encapsulated within Zr-Based Metal–Organic Frameworks as Efficient Heterogeneous Catalysts for Olefins Epoxidation. ACS Sustain. Chem. Eng..

[104] Mohammadi M., Khazaei A., Rezaei A., Huajun Z., Xuwei S. (2019). Ionic-Liquid-Modified Carbon Quantum Dots as a Support for the Immobilization of Tungstate Ions (WO_4_^2–^): Heterogeneous Nanocatalysts for the Oxidation of Alcohols in Water. ACS Sustain. Chem. Eng..

[105] Jiménez-González C., Curzons A.D., Constable D.J.C., Cunningham V.L. (2004). Expanding GSK’s Solvent Selection Guide—Application of Life Cycle Assessment to Enhance Solvent Selections. Clean Technol. Environ. Policy.

[106] Wang J.Y., Zhou M.D., Yuan Y.G., Fu N.H., Zang S.L. (2015). Oxidation of Cyclooctene to Suberic Acid Using Perrhenate-Containing Composite Ionic Liquids as Green Catalysts. Russ. J. Gen. Chem..

[107] Zhang J., Wei W.-J., Lu X., Yang H., Chen Z., Liao R.-Z., Yin G. (2017). Nonredox Metal Ions Promoted Olefin Epoxidation by Iron(II) Complexes with H_2_O_2_: DFT Calculations Reveal Multiple Channels for Oxygen Transfer. Inorg. Chem..

[108] Liu C.-G., Jiang M.-X., Su Z.-M. (2017). Computational Study on M1/POM Single-Atom Catalysts (M = Cu, Zn, Ag, and Au; POM = [PW_12_O_40_]^3−^): Metal–Support Interactions and Catalytic Cycle for Alkene Epoxidation. Inorg. Chem..

[109] Kamata K., Kotani M., Yamaguchi K., Hikichi S., Mizuno N. (2007). Olefin Epoxidation with Hydrogen Peroxide Catalyzed by Lacunary Polyoxometalate [γ-SiW_10_O_34_(H_2_O)_2_]^4−^. Chem. Eur. J..

[110] Zhao W., Zhang Y., Ma B., Ding Y., Qiu W. (2010). Oxidation of alcohols with hydrogen peroxide in water catalyzed by recyclable keggin-type tungstoborate catalyst. Catal. Commun..

[111] Sutradhar M., Martins L.M., Da Silva M.F.C.G., Pombeiro A.J. (2015). Oxidovanadium complexes with tridentate aroylhydrazone as catalyst precursors for solvent-free microwave-assisted oxidation of alcohols. Appl. Catal. A Gen..

