# Supplementary material for: Organic Solvent-Free Olefins and Alcohols (ep)oxidation Using Recoverable Catalysts Based on [PM12O40]3− (M = Mo or W) Ionically Grafted on Amino Functionalized Silica Nanobeads
Source: Materials (Basel). 2019 Oct 9;12(20):3278. doi: 10.3390/ma12203278 (PMC6829895; doi:10.3390/ma12203278)
Supplement: Supplementary file 1 [file materials-12-03278-s001.pdf]

materials

Supplementary Materials

Organic Solvent-Free Olefins and Alcohols (ep)oxidation Using Recoverable Catalysts Based on $[PM_{12}O_{40}]^{3-}$ (M= Mo or W) Ionically Grafted on Amino Functionalized Silica Nanobeads

Yun Wang [1,2], Florence Gayet [1,3], Pascal Guillo [1,2] and Dominique Agustin [1,2,*]

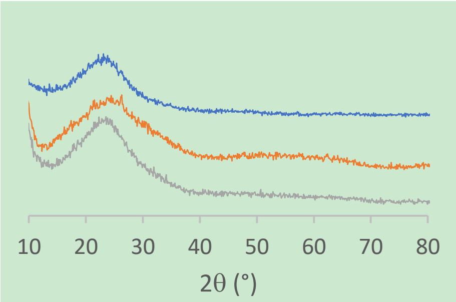

Figure S1. Powder X-ray diffraction diagrams of **SiO_2** (blue), **SiO_2@PW** (orange) and **SiO_2@PMo** (grey) particles.

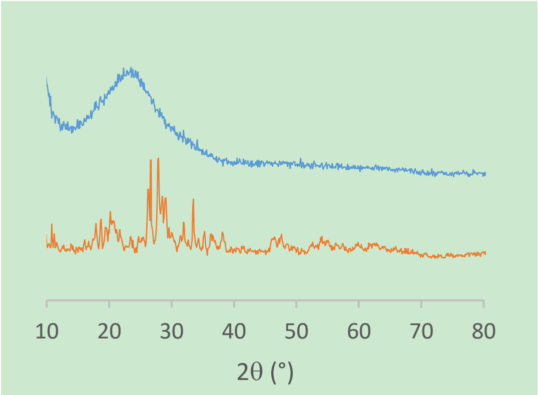

(**a**)

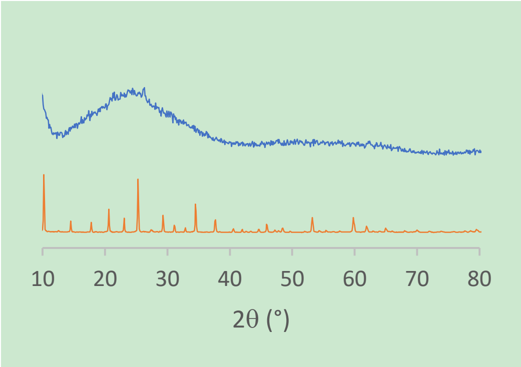

(**b**)

Figure S2. Comparison of Powder X-ray diffractions of (**a**) $H_3PMo_{12}O_{40}$ (orange) and **SiO_2@PMo** (blue) and (**b**) $H_3PW_{12}O_{40}$ (orange) and **SiO_2@PW** (blue). The intensities of **SiO_2@PMo** and **SiO_2@PW** were magnified 10 times.

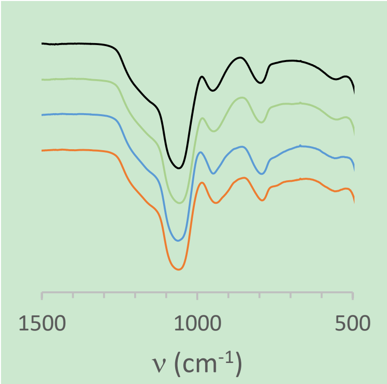

Figure S3. From up to down: Relevant IR vibration zones for **SiO_2, SiO_2@NH_2, SiO_2@PW, SiO_2@PMo.**

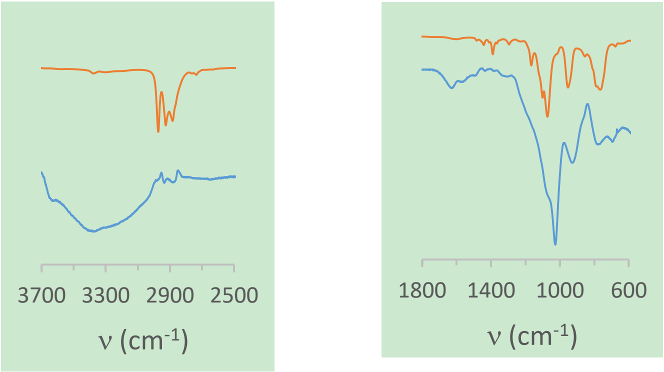

Figure S4. Difference spectra (**SiO_2@NH_2−SiO_2**) on specific ranges (in blue). The spectrum of APTES is indicated in orange.

Table S1. Relevant solid-state NMR data.

	SiO_2	SiO_2@NH_2	SiO_2@PW	SiO_2@PMo
		**[1]H MAS**		
	3.4-	0.9	0.8	0.8
	5.8-	1.2	3.3	3.4
		2.2	4.0	4.0
		3.6	6.5	6.8
		5.1		
		**[13]C CP MAS**		
**CH_2O**		60.4	59.9	59.9
**CH_2O**		58.2	58.2	58.2
**CH_2N**		50.9	50.8	50.9 (
**CH_2N**		42.3	42.8	42.9
**CH_2**		21.5	20.6	20.7
**CH_3**		16.5	16.6	16.6
**CH_2Si**		9.6	9.2	8.8
		**[29]Si CP-MAS (deconvolution is in parenthesis)**		
**T2**		−62.1	−58.3	−58.6
**T3**		−67.7	−67.9	−68.2
**Q2**	−93.3 (7)	−92.8 (6)	−93.0 (7)	−93.0 (7)
**Q3**	−101.9 (49)	−102.0 (57)	−102.1 (67)	−102.1 (66)
**Q4**	−111.8 (44)	−111.5 (37)	−111.7 (26)	−111.7 (27)
		**[29]Si MAS (deconvolution is in parenthesis)**		
**Q2**	−93.5 (4)	−92.4 (7)	−92.6 (9)	−92.8 (9)
**Q3**	−101.9 (32)	−101.8 (37)	−101.9 (33)	−101.9 (27)

**Q4**	−111.8 (64)	−111.6 (56)	−111.7 (58)	−111.6 (64)
		**^{31}P CP MAS (value of free POMs are in parenthesis)**		
			−12.8 (−15.8)	−1.5 (−4.3,−5.0)
		**^{31}P MAS (value of free POMs are in parenthesis)**		
			−13.4 (−15.4,−15.8)	−1.5,−4.7,−6.7 (−4.3)

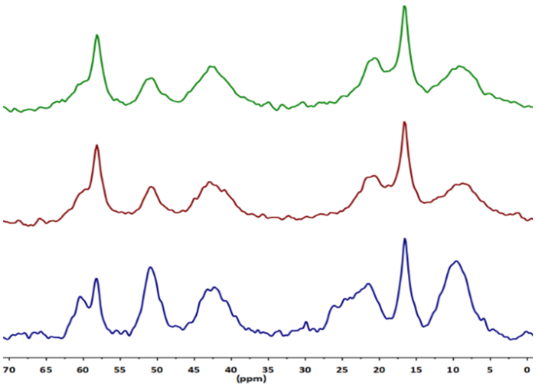

Figure S5. ^{13}C MAS NMR spectra of **SiO_2@PW** (up), **SiO_2@PMo** (middle) and **SiO_2@NH_2** (down).

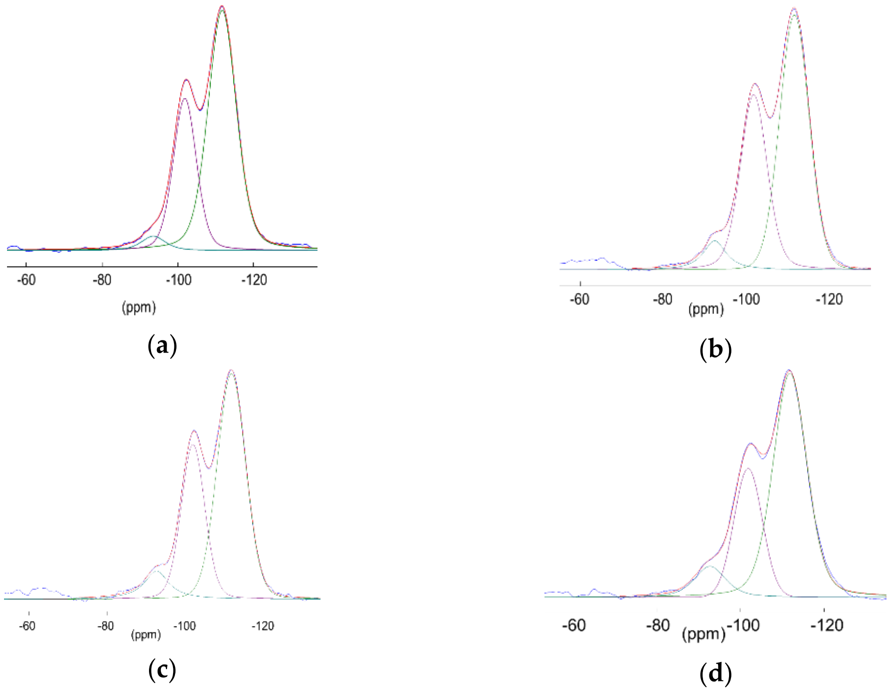

Figure S6. ^{29}Si MAS NMR spectra of **SiO_2** (**a**) **SiO_2@NH_2** (**b**), **SiO_2@PW** (**c**) and **SiO_2@PMo** (**d**).

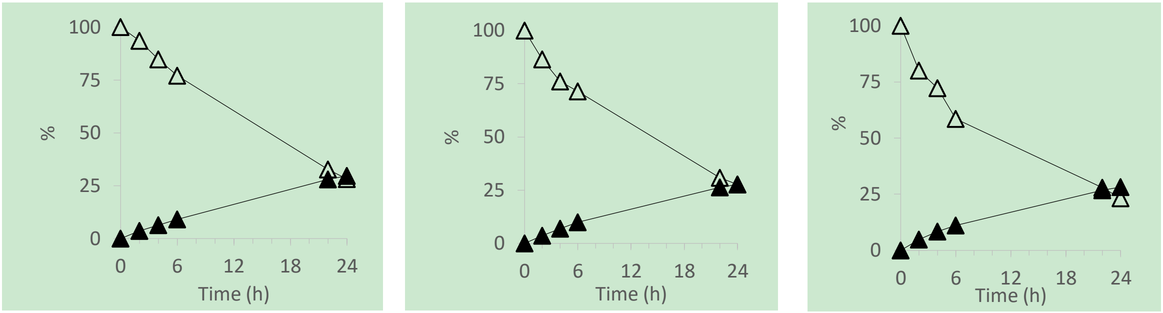

(**a**) (**b**) (**c**)

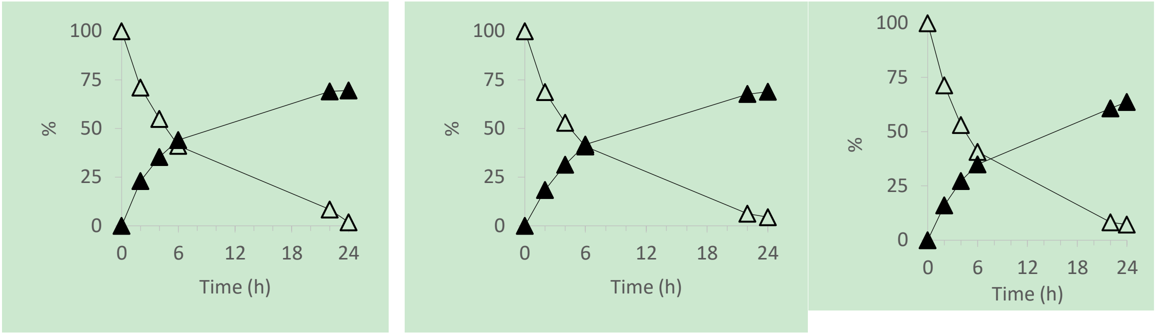

(**d**) (**e**) (**f**)

Figure S7. Evolution of CO (△) and COE (▲) with **SiO₂@PW** (Run 1 (**a**), Run 2 (**b**) and Run 3 (**c**)) and **SiO₂@PMo** (Run 1 (**d**), Run 2 (**e**) and Run 3 (**f**))

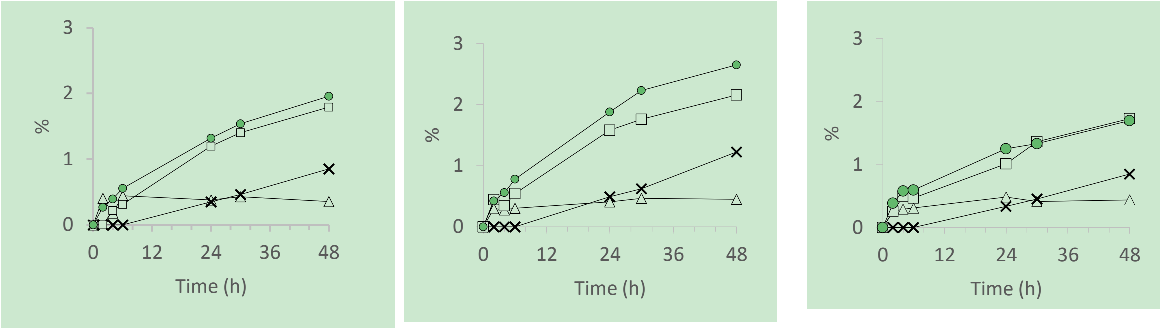

(**a**) (**b**) (**c**)

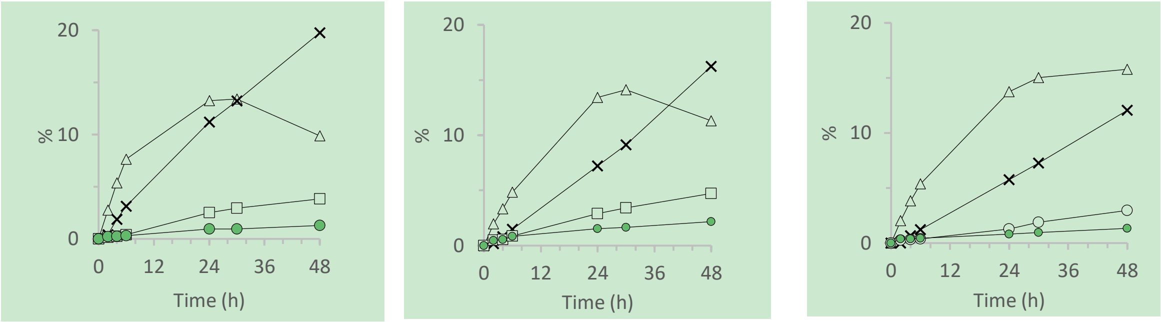

(**d**) (**e**) (**f**)

Figure S8. Evolution of CHO (△), CHD (×), CHol (□) and CHone (●) with **SiO₂@PW** (Run 1 (**a**), Run 2 (**b**) and Run 3 (**c**)) and **SiO₂@PMo** (Run 1 (**d**), Run 2 (**e**) and Run 3 (**f**))

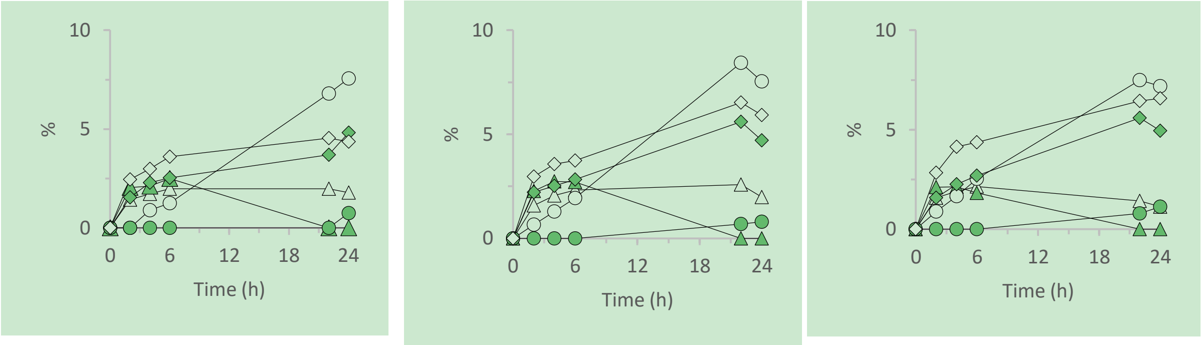

(**a**) (**b**) (**c**)

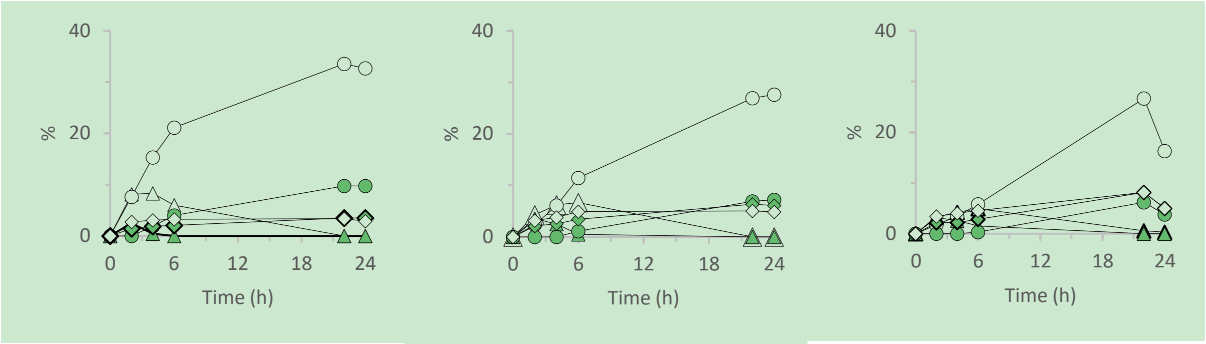

(**d**) (**e**) (**f**)

Figure S9. (**a**) Evolution of *trans*-LO (△), *cis*-LO CHD (▲), eq-LD (●), ax-LD (**O**), C^{ol} (◇) and C^{one} (◆) with **SiO2@PW** (Run 1 (**a**), Run 2 (**b**) and Run 3 (**c**)) and **SiO2@PMo** (Run 1 (**d**), Run 2 (**e**) and Run 3 (**f**))

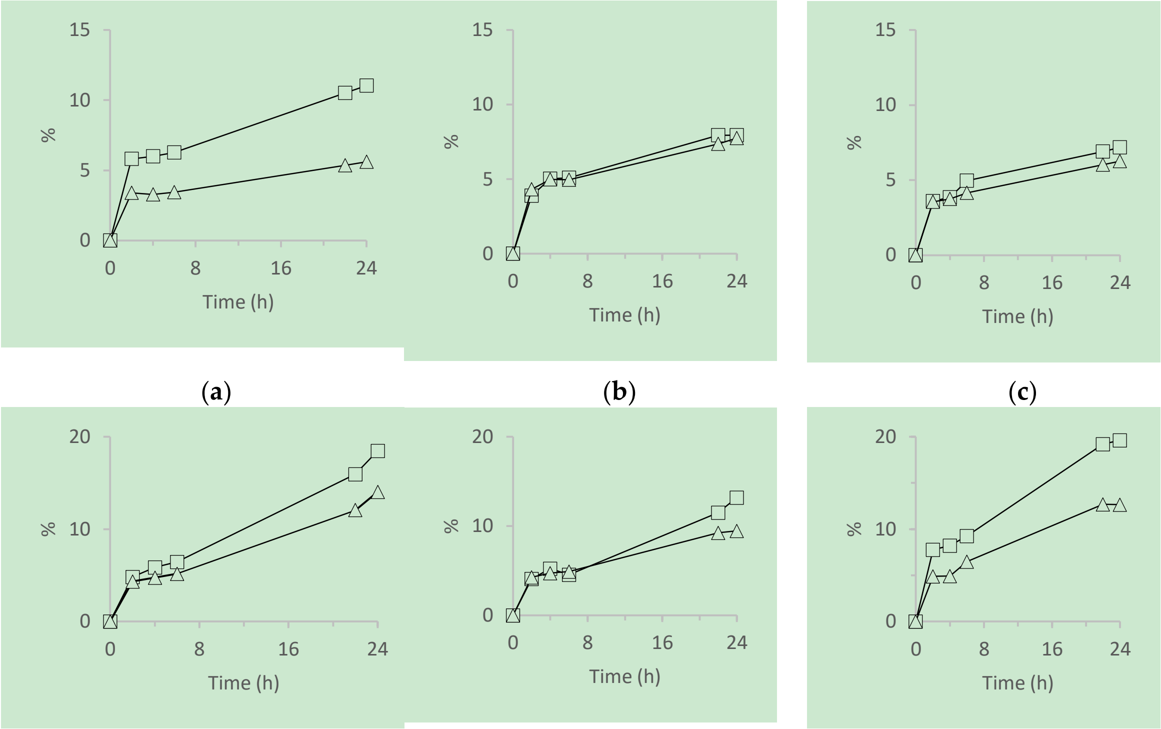

(**d**) (**e**) (**f**)

Figure S10. Conversion of CY^{ol} (□) and formation of CYone (△) with **SiO2@PW** (Run 1 (**a**), Run 2 (**b**) and Run 3 (**c**)) and **SiO2@PMo** (Run 1 (**d**), Run 2 (**e**) and Run 3 (**f**))
